# A folding motif formed with an expanded genetic alphabet

**DOI:** 10.1038/s41557-024-01552-7

**Published:** 2024-06-10

**Authors:** Bang Wang, James R. Rocca, Shuichi Hoshika, Cen Chen, Zunyi Yang, Reza Esmaeeli, Jianguo Wang, Xiaoshu Pan, Jianrong Lu, Kevin K. Wang, Y. Charles Cao, Weihong Tan, Steven A. Benner

**Affiliations:** 1 Zhejiang Cancer Hospital, The Key Laboratory of Zhejiang Province for Aptamers and Theranostics, Hangzhou Institute of Medicine (HIM), Chinese Academy of Sciences, Hangzhou, China; 2 Center for Research at Bio/Nano Interface, Department of Chemistry, Department of Physiology and Functional Genomics, Health Cancer Center, University of Florida, Gainesville, FL USA; 3 AMRIS, McKnight Brain Institute, University of Florida, Gainesville, FL USA; 4 Foundation for Applied Molecular Evolution, Alachua, FL USA; 5 Firebird Biomolecular Sciences LLC, Alachua, FL USA; 6 College of Chemistry and Chemical Engineering, Inner Mongolia Key Laboratory of Fine Organic Synthesis, Inner Mongolia University, Hohhot, China; 7 Department of Biochemistry and Molecular Biology, College of Medicine, University of Florida, Gainesville, FL USA; 8 Department of Emergency Medicine, University of Florida, Gainesville, FL USA; 9 Institute of Molecular Medicine (IMM), Renji Hospital, Shanghai Jiao Tong University School of Medicine, College of Chemistry and Chemical Engineering, Shanghai Jiao Tong University, Shanghai, China; 10 Molecular Science and Biomedicine Laboratory (MBL), State Key Laboratory of Chemo/Biosensing and Chemometrics, College of Chemistry and Chemical Engineering, College of Biology, Aptamer Engineering Center of Hunan Province, Hunan University, Changsha, China

**Keywords:** DNA, DNA, Synthetic biology

## Abstract

Adding synthetic nucleotides to DNA increases the linear information density of DNA molecules. Here we report that it also can increase the diversity of their three-dimensional folds. Specifically, an additional nucleotide (dZ, with a 5-nitro-6-aminopyridone nucleobase), placed at twelve sites in a 23-nucleotides-long DNA strand, creates a fairly stable unimolecular structure (that is, the folded Z-motif, or fZ-motif) that melts at 66.5 °C at pH 8.5. Spectroscopic, gel and two-dimensional NMR analyses show that the folded Z-motif is held together by six reverse skinny dZ^−^:dZ base pairs, analogous to the crystal structure of the free heterocycle. Fluorescence tagging shows that the dZ^−^:dZ pairs join parallel strands in a four-stranded compact down–up–down–up fold. These have two possible structures: one with intercalated dZ^−^:dZ base pairs, the second without intercalation. The intercalated structure would resemble the i-motif formed by dC:dC^+^-reversed pairing at pH ≤ 6.5. This fZ-motif may therefore help DNA form compact structures needed for binding and catalysis.

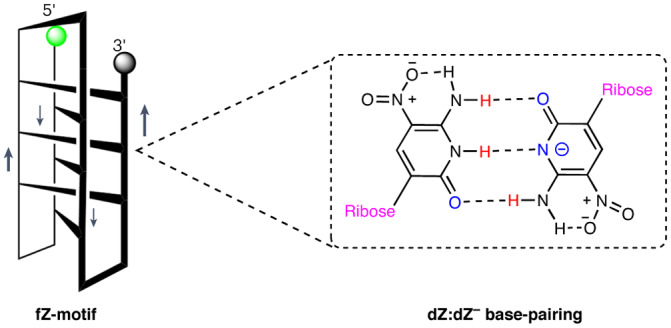

## Abstract (web summary)

Standard DNA is limited by low information density and functional diversity. Now it has been shown that an expanded genetic alphabet—incorporating a synthetic nucleotide, dZ—allows for the creation of stable three-dimensional DNA structures under mild alkaline conditions. Such stable structures enrich our understanding of DNA’s structural diversity and its potential in synthetic biology applications.

## Main

In addition to its well-known double helical forms (A and B) with canonical A:T and G:C pairs, standard DNA can adopt other non-canonical structures that have biological significance, including Z-DNA^1,2^, cruciforms^3^, hairpins^4^ and the adenine motif (A-motif)^5^, which are also (largely) double stranded. More than two strands are seen in triplexes^6^, the four-stranded guanosine-quadruplex (G4)^7^ and two intercalated motifs, the AC-motif^8^ and the i-motif^9^.

The last is a four-stranded DNA structure that assembles via pairs between standard (dC) and protonated (dC^+^) cytidines; each pair carries a positive charge. These are intercalated to form the i-motif. The i-motif plays roles in many biological functions, including maintaining genome integrity and regulating transcriptional activities^10–13^. It is also widely used in sensors and nanoscale motors, which change their conformation with small pH changes^14,15^.

Synthetic biologists have developed alternative forms of DNA that could also support double helical structures by incorporating additional nucleotide letters and thus expanding the genetic alphabet^16^ by rearranging the hydrogen bonding groups that join base pairs together^17^. This makes twelve different nucleotides possible in total, which in turn form six different Watson–Crick nucleotide pairs, many of which have enabled various practical applications within an expanded set of Watson–Crick pairing rules, including diagnostics assays^18^, nanostructures^19^ and aptamers^20–22^.

Like standard DNA, such an expanded genetic alphabet can lead to the formation of non-canonical structures, some of which are entirely inaccessible to standard four-letter DNA. For example, isoguanosine, which forms a Watson–Crick pair with isocytidine, can form pentaplex structures. Its hydrogen bonding patterns and metal coordination abilities are unavailable to standard DNA^23,24^.

**Fig. 1:**
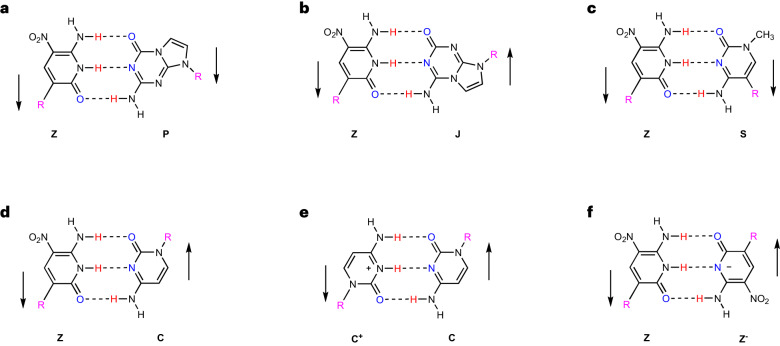
Definition of terms describing pairs between nucleobases in various orientations. **a**, Canonical Watson–Crick pair joining antiparallel strands between dZ and dP, with deoxyribose or ribose (R, labelled pink) oriented down. **b**, Reverse Watson–Crick pair between dZ and dJ, with the 2′-deoxyribose (R) on the left (right) oriented down (up), holding together parallel strands. **c**, Skinny pair between dZ and dS, with both 2′-deoxyriboses (R) oriented down, holding together antiparallel strands. **d**, Reverse skinny pair between dZ and dC; the 2′-deoxyribose (R) on the left (right) is oriented down (up). **e**, Positively charged reverse skinny pair between charge-neutral and protonated C; the 2′-deoxyribose (R) on the left (right) is oriented down (up). **f**, Negatively charged reverse skinny pair between charge-neutral and deprotonated Z; the 2′-deoxyribose (R) on the left (right) is oriented down (up).

Some non-canonical structures with non-standard nucleobases might feature interactions between their protonated and deprotonated forms, analogous to the protonated cytosine required for the i-motif in standard DNA. For example, we recently solved a crystal structure in which 6-amino-5-nitropyrid-2-one (the heterocycle in the added nucleotide known as dZ) crystallizes to give a pair between the neutral aminonitropyridone and the corresponding deprotonated anion, that is, a deprotonated Z pyridone^25^. The negative charges in these crystals are compensated by either ammonium or sodium cations, which coordinate the nitro groups. This pairing was different from the Watson–Crick pair formed by Z in an oligonucleotide with its partner P (containing a 2-amino-8-imidazo-[1,2a]-1,3,5-triazin-[8H]-4-one heterocycle; Fig. 1a)^26^.

The deprotonated Z^−^:Z pairing was surprising because the crystallization was performed at near-neutral pH^25^, which is below the p*K*_a_ of the aminonitropyridone heterocycle (~7.8). Evidently, the stability of the structure drove deprotonation as well as overcoming the repulsion in the packed crystal between pairs with negative charges.

In the context of a duplex formed from two antiparallel strands, the nucleobase in dZ pairs with the nucleobase in dP to give a dZ:dP pair (Fig. 1a)^27^. Featuring both size and hydrogen bonding complementarity, this pair is analogous to the standard C:G pair.

We first questioned whether dZ^−^:dZ pairs might form in the context of an oligonucleotide fold. The deprotonated Z^−^:Z pair observed in the previously reported crystal structure^25^ differs from the canonical Watson–Crick pair in several ways. First, it is skinny, matching a small pyrimidine with another small pyrimidine (Fig. 1c–f); this has been previously seen in antiparallel helices^28^. Second, the deprotonated Z^−^:Z pair involves reverse pairing, with one ribose (R) oriented up and the other oriented down (Fig. 1b,d,e,f). Reverse pairing is generally seen to hold together parallel strands in size-complementary purine:pyrimidine matches^29,30^.

In many respects, a deprotonated dZ^−^:dZ pair resembles the dC:dC^+^ pairing in the i-motif^31^. Here, the dC:dC^+^ pairing is (1) skinny, (2) reverse and (3) charged, albeit positively charged (the deprotonated Z^−^:Z pair is negatively charged).

Recognizing that the deprotonated Z^−^:Z pairing might allow non-canonical structures in a non-canonical fold with an expanded DNA alphabet, we herein synthesized oligonucleotides that might form multiple deprotonated dZ^−^:dZ pairs (Table 1). We also synthesized several control sequences that might disrupt folded structures that feature dZ^−^:dZ pairing, including a dZ-rich molecule with a random sequence. We also synthesized sequences that form the classical dC:dC^+^ i-motif, and a dC-rich control with the same nucleotides in random order.

**Table 1:** Oligonucleotides and the sequences used in this study

Name	Sequence (5′ to 3′)
i-Motif	ACCCTAACCCTAACCCTAACCCT
C-control	ACTCACACACCACCTCTCACTCT
ZZZ (fZ-motif)	AZZZTAAZZZTAAZZZTAAZZZT
Z-control1	AZTZAZAZAZZAZZTZTZAZTZT
Z-control2	AZZZTAATTTTAATTTTAAZZZT
Z-control3	AZZZTAATTTTAAZZZTAAZZZT
i-Motif-FQ	FAM-ACCCTAACCCTAACCCTAACCCT-Dabcyl
C-control-FQ	FAM-ACTCACACACCACCTCTCACTCT-Dabcyl
ZZZ-FQ	FAM-AZZZTAAZZZTAAZZZTAAZZZT-Dabcyl
Z-control4-FQ	FAM-AZZZTAACCCTAAZZZTAACCCT-Dabcyl

We report here data demonstrating that appropriately designed Z-rich structures may adopt a fold with multiple deprotonated dZ^**−**^:dZ pairs under mildly basic (pHs 8–9) conditions. We further discuss whether these structures are derived from analyses of multiple types of experimental data.

## Results

Five different analytical methods were used to evaluate the formation of a folded structure from the synthetic sequences shown in Table 1.

### Thioflavin T assay detection

**Fig. 2:**
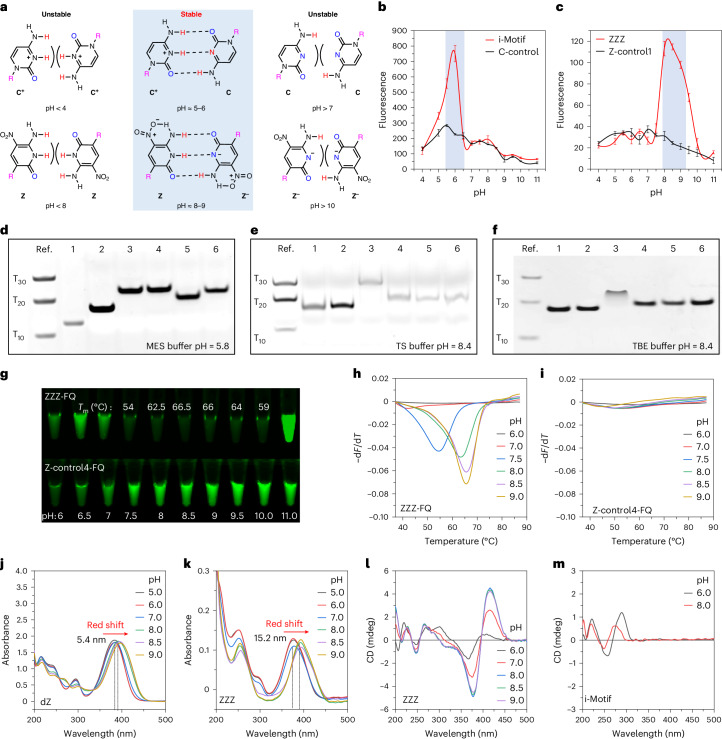
Characterization of fZ-motif. **a**, Structural influence of pH on reverse skinny C:C and Z:Z pair formation in various charge conditions. **b**, The effect of pH on ThT fluorescence when intercalated into C-rich sequences able to form the i-motif via dC:dC^+^ pairing. **c**, The impact of pH on ThT fluorescence when intercalated into dZ-rich sequences able to form the fZ-motif via dZ:dZ^−^ pairing. There were *n* = 5 independent runs; error bars in **b** and **c** represent mean values ± s.d. The blue shaded areas represent the DNA forming the motif structure. **d**–**f**, DNA samples analysed by non-denaturing polyacrylamide gel electrophoresis (20%) in 2-(N-morpholino)ethanesulfonic acid (MES) (**d**), TS (**e**) and TBE (**f**) buffers. Left lane: T_10_, T_20_ and T_30_ oligonucleotides. Lane 1, i-motif; lane 2, C-control; lane 3, fZ-motif; lane 4, Z-control1; lane 5, Z-control2; lane 6, Z-control3. The gels were stained using the Stains-All dye. **g**, Photographs of fluorescence of DNA samples (1 μM) in 100 mM phosphate buffer with pHs ranging from 6 to 11 at 25 °C. **h**,**i**, Melting peaks of ZZZ-FQ (**h**) and Z-control4-FQ (**i**) reveal temperature responses across pHs 6 to 9 in phosphate buffer. **j**,**k**, Absorption spectra of dZ nucleoside (**j**) and ZZZ oligo (**k**) in phosphate buffer across pHs 5–9. **l**,**m**, Circular dichroism (CD) spectra compare ZZZ oligo (**l**) and i-motif (**m**) sequences, highlighting structural insights across pHs 6–9 in phosphate buffer. Source data

The first analytical method exploited the large change in the fluorescence emission spectra of thioflavin T (ThT) when it intercalates into various DNA folds^32^. This allows ThT to be used to probe the formation of the i-motif with dC:dC^+^ skinny, reverse and charged pairs following its transition from a random coil as the pH drops from neutrality to pH 6 and below, at which point it forms (Fig. 2a).

With the ZZZ sequence, fluorescence arising from bound ThT was observed at pH ≈ 8–9.5, but not at higher or lower pHs (Fig. 2c). The maximum fluorescence is seen at pHs 8–9, which is consistent with the formation of a folded Z-motif (fZ-motif), with the pH range of formation suggesting that it involves a deprotonated dZ^−^:dZ pairing (Fig. 2a).

This analysis included three controls. First, consistent with the literature^33^, the sequence designed to fold into a C:C^+^ i-motif also fluoresced, but over a lower pH range of ~5–6 (Fig. 2b); this is the range in which protonation on cytosine is permitted. This pH range is higher than expected from the p*K*_a_ of cytosine, presumably due to the intrinsic stability of the fold. Furthermore, a C-rich control molecule with a randomized sequence showed no intercalation of ThT; this is also consistent with the literature^33^ (Fig. 2b).

A Z-control1 sequence featuring the same composition—but a different sequence that is unable to form the proposed motif—showed no comparable changes in fluorescence; it evidently does not fold into a form that is able to bind ThT. Likewise, a C-rich DNA molecule with the same C nucleotides as in the C:C^+^ i-motif itself, but in a randomized order, did not exhibit fluorescence. Quinaldine red^34^—a fluorescent molecule used to detect the i-motif fold—was also tested, and a similar phenomenon was observed (Supplementary Fig. 1).

### Gel shift analysis

Although the fluorescence of thioflavin T and some other dyes are routinely used to study the folding of standard DNA sequences, including those with skinny reverse charged pairs, we recognized that the intercalation of the dye may perturb the molecular structure of the dZ-rich folds. Accordingly, we applied several other analytical tools.

The first relied on the fact that folded DNA migrates differently in an electrical field than unfolded DNA. Thus, the electrophoretic mobilities of a series of oligodeoxynucleotides were compared via non-denaturing polyacrylamide gel electrophoresis. At pH 5.8, the classical C-rich i-motif sequence DNA (lane 1) migrates faster than the C-rich randomized sequence (lane 2) (Fig. 2d). The faster mobility of the i-motif has been reported in the literature^35,36^. By contrast, at pH 8.4, where essentially none of the cytidine is protonated and no C:C^+^ pairs form, the i-motif sequence migrates at the same rate as the randomized control (Fig. 2e,f). Again, from the literature, this is interpreted as evidence that the classical i-motif structure is not formed at higher pHs. This interpretation is robust with respect to changing the buffer from Tris-H_2_SO_4_ (TS) to Tris-Borate-EDTA (TBE). The classical i-motif runs faster when folded.

Analogous results were seen with the dZ-rich target sequence. The ZZZ sequence is not expected to fold into an fZ-motif at low pHs (Fig. 2a). Consistent with this view, the ZZZ sequence and its randomized control migrate similarly at low pHs. Comparable sequences, with multiple thymidine’s replacing the dZs, migrate differently. This is seen generally in the electrophoresis of single-stranded DNA; adding dZs to an oligonucleotide slows its migration.

At higher pHs, at which the fZ-motif is expected to form, the target dZ-rich sequence migrates slower than the analogous molecule with a randomized sequence, and slower than molecules where the fZ-motif would be disrupted by replacement of Z by thymidines. Interestingly, the fZ-motif runs slower when folded at pH 8.4; this is 0.6 pH units higher than the pK_a_ of Z, implying greater charge. Further, the unfolded molecule at higher pH would have more negative charges—approximately four more than in the folded form, and the folded fZ-motif structure could potentially protect the negative charge at the centre of the motif. This may explain the mobility difference.

### Fluorescence quenching assessment of folding

To obtain further information on the fZ-motif structure, various DNA molecules were synthesized with a fluorescent tag (fluorescein, FAM) at their 5′-ends and a quencher (Dabcyl) at their 3′-ends (Table 1). Their fluorescence was then examined as a function of pH. The FAM probe cannot be used at low pHs because its chromophore is lost^37^ below pH ≈ 6.5. Nevertheless, substantial differences were seen with the classical dC:dC^+^ i-motif and dC-rich random sequences at pHs 6 and 5.5 (Supplementary Fig. 2). Specifically, folding of the classical dC:dC^+^ i-motif sample loses fluorescence compared with the C-random sequence. This is consistent with i-motif formation below pH ≈ 7, as previously reported^33^.

The loss of fluorescein fluorescence was not relevant to the interpretation of the dZ-rich sequence because the fZ-motif does not form at low pH. With the fZ-motif, fluorescence was absent between pH ≈ 7.5 and pH ≈ 10. This is attributed to the formation of the fZ-motif, which brings the FAM and quencher into close proximity (Fig. 2g); however, fluorescence returns at pHs above 10, which is consistent with full deprotonation of dZ, the consequent loss of the possibility of dZ^−^:dZ skinny reverse pairs (Fig. 2a) and the unfolding of the fZ-motif. This also provides our first piece of conformational information. Whichever fold is formed at intermediate pHs, the fZ-motif must bring the 5′-end of the molecule near to the 3′-end at pHs ≈ 7.5–9.5.

The melting temperatures of these folded structures were measured by using fluorescence as a probe. The classical C:C^+^ i-motif melted at ~58 °C at pH 5.5 (Supplementary Fig. 2). The deprotonated dZ^−^:dZ fZ-motif melted at ~66.5 °C at pH 8.5 (Fig. 2h and Supplementary Fig. 3). The Z-control sequence (where the fZ-motif was disrupted by introducing cytidine) showed fluorescence at all pHs above 6.5; fluorescein itself becomes protonated and loses fluorescence below pH 6.5. As the Z-control4-FQ does not fold, the *T*_m_ of its folding cannot be measured for the control sequence (Fig. 2g, bottom, and 2i).

Concentration studies were performed to establish whether the fold is unimolecular. With triplicate runs, the melting temperature—as determined by fluorescence quenching—was unchanged across oligo concentrations of 15 nM to 2,000 nM (the data are summarized in Supplementary Figs. 4 and 5). Interestingly, Mg^2+^ (Supplementary Fig. 6) was found to destabilize the fold at >8 mM, whereas the addition of NaCl (Supplementary Fig. 7) stabilized it.

### Ultraviolet–visible and circular dichroism absorption spectroscopy

Ultraviolet–visible molecular absorption spectroscopy was also used as a probe to observe the formation of the fZ-motif. Across pHs 5–9, the absorbance of ZZZ shifts from ~375 nm to ~395 nm with a small amount of hyperchromicity (~5%) (Fig. 2k). The shift (15.2 nm) was most notable between pHs 7 and 8 as increasing numbers of Z nucleobases are deprotonated. This is larger than the red shift (5.4 nm) seen with isolated single dZ nucleotides upon simple deprotonation (Fig. 2j), and may reflect folding as well as simple deprotonation.

The characteristic features of the circular dichroism spectrum of the classical i-motif structure include a strong positive band at ~290 nm and a negative band at ~260 nm (Fig. 2m). This corresponds to the wavelengths at which natural DNA absorbs. The circular dichroism spectrum changes characteristically with changing pH as the i-motif forms, reflecting a large conformational change that forms the fold.

For the untagged ZZZ sequence, the circular dichroism spectrum is very different from the untagged standard sequence. This is expected because the ultraviolet absorbance properties of dZ are very different from those of standard DNA. In particular, the Z heterocycle absorbs between 350 and 400 nm, where standard DNA does not. Thus, the circular dichroism spectra of dZ-containing oligonucleotides has features at this wavelength. Consistent with the fZ-motif forming at higher pHs, the circular dichroism spectra of the Z-rich target sequence changed most dramatically between pHs 7 and 8 (Fig. 2l). Here, again, the circular dichroism signal changes upon changing the pH from 7 to 8. Circular dichroism spectroscopy is thus a further probe of the formation of the fZ fold at these pHs.

### Nuclear magnetic resonance spectra

To further characterize the structure formed by a ZZZ sequence, a solution of ZZZ oligonucleotide (2 mM) was examined by NMR (800 MHz Bruker) at pH 7 in D_2_O, and at pH 8.5 in both D_2_O and a 9:1 mixture of H_2_O/D_2_O. In D_2_O, signals from exchangeable protons attached to the oligonucleotide were lost, but all of the other protons were observed. In the 9:1 H_2_O/D_2_O solution, the signals from solvent protons (4.5–5 ppm) were suppressed, allowing observation of signals assigned to the exchangeable protons, in particular, the NH_2_ and N–H protons on the ring heterocycles.

**Fig. 3:**
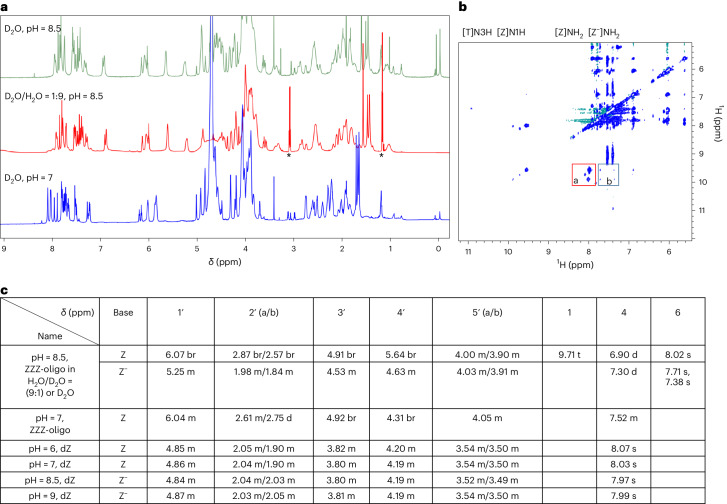
Characterization of fZ-motif structure by NMR. **a**, ^1^H NMR spectra of ZZZ oligo at indicated pH in two indicated solvent systems. All samples contain 50 mM phosphate buffer. The asterisks represent DNA samples from which triethylamine was not fully removed by desalting. **b**, NOESY spectrum of ZZZ at pH = 8.5 in H_2_O/D_2_O (9:1). Box a (red) holds the cross-peak assigned to neighbouring [Z]N1H and [Z]NH_2_. Box b (blue) holds the cross-peak assigned to neighbouring [Z]N1H and [Z^-^]NH_2_. **c**, ^1^H NMR spectrum assignment of ZZZ at pH = 8.5 in D_2_O solution or in H_2_O/D_2_O (9:1), ZZZ at pH = 7 in D_2_O solution and free dZ over a range of pHs (6, 7, 8.5 and 9 in D_2_O). The primes indicate protons in sugar. Signal multiplicity is abbreviated as follows: s, singlet; d, doublet; t, triplet; m, multiplet; and br, broad.

As the ZZZ molecule has only three different building blocks (dT, dA and dZ), inadequate dispersion did not allow full assignment by walking down the chain, even at 800 MHz. However, spin systems could be assigned. In particular, in D_2_O at pH 8.5, H–H correlation spectroscopy (COSY), total correlation spectroscopy (TOCSY) and nuclear Overhauser enhancement spectroscopy (NOESY) assigned all of the protons to their individual dA, dT, dZ and dZ^−^ nucleotide spin systems. This included signals from the thymidine methyl protons, which were connected to their partner 6-position proton signals by COSY. Further intra-nucleotide NOESY allowed signals from the sugar protons to be connected with their bases. In particular, we connected the ring T (6), ring A (8 or 2) and ring Z (4) protons to their respective 2′-deoxyribose spin systems (Fig. 3a and Supplementary Figs. 10, 12 and 14).

We carefully studied the changes in the spectra upon going from pHs 7 (unfolded) to 8 (folded) to identify characteristic signals and fingerprints associated with the folding of the fZ-motif. We first examined the impact of changing the pH from 6 to 9 on the free dZ nucleoside in solution to obtain a baseline (Fig. 3b). Here the pH influences the chemical shifts only modestly. For example, the chemical shift of the aromatic 4-position proton of free dZ nucleoside moves slightly, from 8.07 to 7.99, upon deprotonation. The chemical shift of the sugar protons of dZ moves even less.

The chemical shifts of the protons of dZ nucleotides embedded in the oligonucleotide differ from those in the free nucleotide, even at pH 7, at which the oligonucleotide is largely unfolded. Figure 3c captures these differences. Some of the differences, in particular, the downfield movements of the 3′-protons (from 3.8 in the free dZ to 4.8 in the dZ in the unfolded oligonucleotide) and the 5′-protons (from 3.5 to 4) simply reflect the esterification of the R–OH unit (in the nucleoside) to a phosphate (in the oligonucleotide). Not attributable to this is the large downfield movement of the 1′-protons (from ~4.8 to ~6.0) and the upfield movement of the base-4 protons (from ~8.0 to ~7.5). This is undoubtedly due to changes in the local environments in the oligonucleotide relative to the free nucleoside.

Further changes were seen in the proton NMR following folding at pH 8.5; these were diagnostic for the formation of the fZ-motif. The most dramatic change upon forming the fZ-motif is seen in the Z-4 protons. At pH 7, the twelve protons arising from the twelve dZ nucleotides resonate around 7.5 ppm. This is near the 8.0 chemical shift seen for the Z-4 proton in the isolated nucleoside. However, following formation of the fZ-motif, the Z-4 protons split into two groups, each with six members. The six protons in one group resonate dramatically upfield from their signals in the unfolded oligonucleotide, with chemical shifts 6.92–6.95 ppm. The other six have chemical shifts at 7.29–7.32 ppm. This is consistent with a fZ-motif containing six dZ^−^:dZ pairs. We tentatively assign the dZs whose N-4 signal shifts upfield as the dZ^−^ partner in the dZ^−^:dZ pairs. The failure of the two forms of dZ to interconvert rapidly on the NMR time scale may reflect constraints on the structure overall, including the placement of cations in the system.

Large shifts are also seen in the sugar protons of dZ as the motif forms (Fig. 3c). The Z-1′ protons in dZs tentatively assigned as dZ^−^ (on the basis of the NOESY spin system assignments) move upfield from 6 to 5.25. Both of the 2′-protons in the dZ^−^ units also move upfield. The same protons in the spin system arising from the neutral dZ move less. Again, this is consistent with a fZ-motif containing six dZ^−^:dZ pairs.

The same pattern applies to the 3′-protons, except that the shifts observed upon folding are smaller. Thus, the 1′-protons from the dZs tentatively assigned as neutral dZ units scarcely move at all following folding. With the 4′-protons, folding into the fZ-motif moves the neutral dZ protons downfield by ~1.3 ppm, with the dZ^−^ protons less perturbed. Only the 5′-protons behave similarly in the dZ^−^ and dZ units in the folded fZ-motif.

We then sought to identify signals and cross peaks arising from the exchangeable protons, which are lost in D_2_O. The spectra were taken in a 9:1 mixture of H_2_O and D_2_O. As expected, broad resonances appeared at 8.5–10 ppm. These are expected to include the six bridging protons in the six dZ^−^:dZ pairs; the –NH_2_ protons on both the protonated and deprotonated dZs, as well as those in the H_2_O/D_2_O (9:1) solution; strong cross-peaks between resonances 9.5 and 10 ppm (N1H of Z), and resonances in the 8–8.1 ppm range (NH_2_ of Z) were found (Fig. 3b, box a). These may identify [Z]N1H protons that are close to [Z]NH_2_ protons. The weaker cross-peaks between resonances at 9.5–10 ppm (N1H of Z) and resonances at 7.38 and 7.71 ppm (NH_2_ of Z^−^) were also found (Fig. 3b, box b). These may identify [Z]N1H and [Z^−^]NH_2_ pairs that are near in space. These features may be characteristic of dZ**:** dZ^−^ base pairs in the fZ-motif structure.

### Metal coordination

**Fig. 4:**
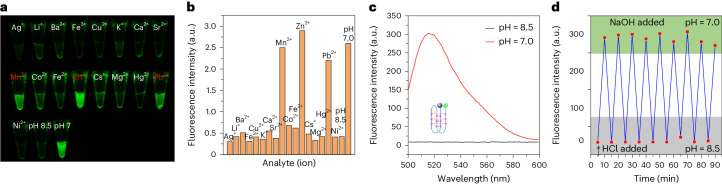
Using fZ-Motif as a sensor. **a**, Photographs of ZZZ-FQ samples (1 µM) with various metal ion species added (5 mM) at pH 8.5 (50 mM phosphate buffer) at 25 °C (incubated overnight). The red-highlighted ions represent the ions that disrupt the DNA fold. **b**, Quantitation of the fluorescence using a quantitative PCR instrument. In several cases, precipitates of the metal phosphates were seen, lowering the effective metal ion concentration. **c**, Fluorescence emission spectra at 25 °C of the ZZZ oligonucleotide as it is reversibly converted from its closed folded state (at pH 8.5) to its open unfolded state (pH 7). Excitation was performed at 488 nm (1 µM DNA sample in 50 mM phosphate buffer at pH 8.5 or 7). **d**, Cycle system of fZ-motif was monitored by fluorescence spectroscopy with excitation at 488 nm and emission at 517 nm. Source data

We further tested the effect of metal cations on the folding of the fZ-motif by examining the change in fluorescence quenching of FAM in the presence of various metal ions at pH = 8.5 (Fig. 4a,b). Many metal ions stabilize nucleic acid secondary structures^38,39^. However, in the fZ-motif system, Mn^2+^, Zn^2+^ and Pb^2+^ seem to disrupt the fold, as indicated by the appearance of fluorescence in their presence. The fZ-motif may offer a tool to detect Mn^2+^, Zn^2+^ and Pb^2+^ ions.

### Use of the fZ-motif in a sensor

DNA nanomachines based on the classical i-motif under slightly acidic conditions have been reported^14^. These exploit the disruption of the i-motif fold upon deprotonation of the dC:dC^+^ pair at higher pH. That disruption is observed by fluorescence spectroscopy with tagged molecules.

To determine whether similar behaviour can be observed at higher pHs with a fZ-motif, we performed an analogous experiment in which the formation and disruption of the fZ-motif was driven by pH oscillations between pHs 7 and 8.5. The ZZZ-FQ sequence were used. At pH 8.5, when the 5′- and 3′-ends of ZZZ are nearby in the fold, the fluorescence of FAM is quenched by Dabcyl. Fluorescence is strong at pH 7, when unfolding of the fZ-motif allows FAM to move away from Dabcyl (Fig. 4c). The fluorescence of this system therefore depends on whether the machine is open or closed. Multiple cycling of the machine was demonstrated by alternating addition of HCl and NaOH. Figure 4d shows the cyclical changes in fluorescence emission that result from controlled opening and closing of the system.

### Density functional theory calculations

Preliminary density functional theory calculations were performed to analyse possible pairing in the fZ-motif (Supplementary Figs. 8 and 9). These calculations started with the geometry of the Z^−^:Z pairing observed in the structure of the crystal formed from the aminonitropyridone heterocycle alone^25^.

The Gibbs free energy of the hypothetic Z**:**Z pair decreased by 1.93 kcal mol^–1^ due to the partial separation of electron distribution in its HOMO and LUMO. However, under alkaline conditions, Z can convert into a Z^−^ anion, leading to complete separation of electron cloud distribution in the HOMO and LUMO of the Z^−^**:**Z base pair. This separation, combined with the formation of three hydrogen bonds, results in a substantial reduction of 346.46 kcal mol^–1^ in the Gibbs free energy of the Z^−^**:**Z base pair, contrasting with the modest reduction of 49.19 kcal mol^–1^ in the Gibbs free energy of the C^+^:C (Supplementary Figs. 10–12). This may also result the Z**:**Z^−^ pair being more stable than the natural pair^40,41^.

## Discussion

Synthetic biologists seek to understand the diversity of possible informational molecules that might support life, including linear polymers that may support genetics in alien life on other worlds. Thus, synthetic biologists have now explored the canonical double helical anti-parallel structures that different forms of DNA can adopt in some detail, demonstrating that many alien genetic systems are possible in the cosmos.

In contrast, analysis of non-canonical folds available to such alien genetic systems has only begun. Just as Terran DNA uses non-canonical structures that are available intrinsically to DNA built from the four canonical nucleotides (GACT or GACU) in DNA and RNA in our biology, alien life might be expected to exploit non-canonical structures that are intrinsic to their different informational polymers throughout their biology.

We show here the formation of one of these non-canonical single-strand folded structures for a set of nucleotide elements chosen from an artificially expanded genetic information system. Although a completely resolved NMR three-dimensional structure is not yet available, we can be confident that the fZ-motif exists, and that it is supported by dZ^−^**:**dZ pairing.

Fluorescence studies establish that in the fold, the 5′- and 3′-ends are in close proximity. Furthermore, dZ^**−**^**:**dZ pairing must have a reverse geometry, as it is the only geometry that allows three hydrogen bonds to be formed; these three bonds are necessary to account for the stability of the fold. That stability at the optimal pH is much greater than the stability of the analogous folded i-motif with its reverse C^+^:C pairs, even at its optimal pH.

**Fig. 5:**
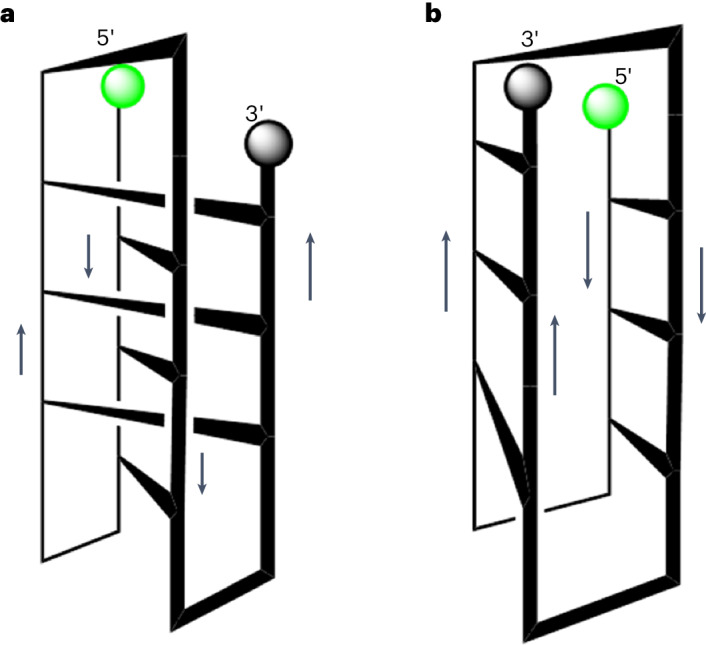
Two down–up–down–up topologies possible for the fZ-motif. **a**, Topology 1: a motif structure with intercalated pairs, as in the i-motif with skinny reverse C^+^:C pairs. **b**, Topology 2: a motif structure with non-intercalated edge-on pairs.

Reverse pairs imply that parallel strands contribute dZ^−^ and dZ nucleotide pairs. This implication is taken from an analogy with the i-motif with skinny reverse C^+^:C pairs. This has a down–up–down–up strand sequence to allow two sets of charged pairs to bring the 3′-end back together with the 5′-end. This, in turn, constrains the fZ-motif to one of two similar topologies, shown in Fig. 5.

- Topology 1: analogous to the topology of the i-motif with its reverse C^+^:C pairs, has a down–up–down–up structure holds parallel strands stabilized by dZ^−^**:**dZ pairs that are intercalated.
- Topology 2: an entirely novel fold in which the down–up–down–up structure holds parallel strands stabilized by dZ^−^**:**dZ pairs that are not intercalated, but rather lie alongside of each other.

Favouring topology 1 is perhaps the precedent provided by the classical i-motif. It also includes the possibility of stabilizing *π*–*π* stacking interactions between the aminonitropyridone aromatic systems. Such interactions may be reflected in crystal structures of canonical double helices that incorporate multiple adjacent dZ:dP pairs^26^. Disfavouring it is the expected repulsion between the negative charges that are carried by each of the stacked pairs.

Interestingly, theoretical studies by Šponer and colleagues have noted similar issues with the classical i-motif^42^. These studies estimate perhaps 65 kcal mol^–1^ of electrostatic destabilization arising from the close proximity of multiple reverse C^+^:C pairs. These might be expected to prevent the formation of the classical i-motif. With the classical C^+^:C i-motif, the positive charges may be compensated, at least in bulk, by the negative charges on the phosphate backbone. No such compensation is possible with the fZ-motif.

In our studies, the most likely counter-ion available to compensate for the negative charges (phosphate and base) is sodium. The fact that the Z and Z^−^ bases are distinct and slowly interconverting on the two-dimensional NMR time-scale suggests that the sodium counter-ions are fixed at sites of the structure.

These considerations notwithstanding, these studies show that such motifs do form with alien genetic systems. Thus, it opens the door not only to addressing the theoretical and stability issues addressed in the paragraph above, but also the use of these in nanostructures that have practical value as nanomachines, signalling architectures and medical devices. Due to its pH driven reversible ‘on/off’ activity, the molecule can trigger the conformational change in slightly alkaline conditions, complementing the i-motif, which triggers in slightly acidic conditions. Although these are not machines in the normal sense of the term, a combination of fZ-motif and i-motif by artful design may be useful in signalling and, in the future, in the field of DNA logic circuit design.

In any case, the fZ-motif adds to the repertoire of folded structures that may enable the evolution of Artificially Expanded Genetic Information Systems (AEGIS)-based catalysis. Such folded structures continue to be explored in natural DNA and RNA to understand catalysis that is possible in canonical systems^43^.

## Methods

### Detection of folding using fluorescent ThT

DNA oligomers (1 µM) and ThT (6 µM) were prepared in 100 mM Tris-HCl buffer at pH 4–11 at 25 °C. The solutions were incubated overnight at room temperature. The fluorescent data were measured using Greiner Bio-One 96-well micro-plates by a Biotek Synergy 2 microplate reader. The excitation and emission filters were 450/15 nm and 490/15 nm, respectively. Error bars are s.d. (*n* = 5).

### Ultraviolet–visible absorption spectroscopy

Ultraviolet–visible absorption spectra were obtained using a NanoDrop spectrophotometer (Thermo Fisher Scientific). DNA samples (10 µM) or dZ (1 mM) were diluted in 50 mM phosphate buffer at various pHs and incubated overnight at 25 °C. Readings were taken between 200 and 500 nm.

### Circular dichroism measurements

Circular dichroism experiments were performed using a Jasco-810 instrument. DNA samples were diluted to 1 µM in 50 mM phosphate buffer at various pHs and incubated overnight at 25 °C. Measurements were performed at wavelengths between 200 and 500 nm, with 1 nm steps and a 1 s response time at 25 °C. The circular dichroism spectra show the average of three scans of the same sample after baseline correction.

### Gel shift experiments

All DNA samples were incubated at pH 5.8 or pH 8.4 in 100 mM Tris-HCl buffer at room temperature overnight before being loaded into the gel. Native gel loading buffer (10X) was added with mixing. The DNA samples were analysed via non-denaturing polyacrylamide gel electrophoresis (20% MES-PAGE; TS-PAGE or TBE-PAGE) at 4 °C. Gels were stained using the Stains-All dye (following instructions) and scanned by a Typhoon Imaging System at Cy5 channel (Amersham Biosciences).

### Fluorescence quenching assessment of folding analysis

Oligonucleotides were modified by attaching FAM fluorescein at the 5′-end and Dabcyl quencher at the 3′-end. DNA samples were diluted to 1 µM in 100 mM phosphate buffer at pHs 5–11, with incubation overnight at 25 °C. Images at different pH values were obtained via photography in a gel-image box under ultraviolet light (365 nm).

### Thermal melting analysis

DNA samples were diluted to 1 µM in 100 mM phosphate buffer at various pHs, pre-heated to 90 °C (30 s), and then cooled down and left to incubate overnight at 25 °C. The melting curves were analysed by visualizing FAM fluorescence in a Roche LightCycler 480 with the following temperature profile: to obtain the melting curve, the sample was heated to 37 °C and held at that temperature for 2 min; the sample was then denatured by heating it from 37 °C to 90 °C, with a melting setting of 5 °C min^–1^; the sample was then cooled to 37 °C.

For the cooling curve, the sample was denatured at 90 °C and held at that temperature for 10 s; the sample was then cooled from 75 °C to 37 °C, with a cooling setting of 2.4 °C min^–1^. All samples were measured three times, and these measurements were run in parallel on a 96-well plate; *T*_m_ values were obtained from the denaturing ramps by using the automatic calculation method in the Roche LightCycler (Melt Factor set at 1.2, Quant Factor set at 20).

### Density functional theory calculations

The molecular orbital amplitude plots of the HOMOs and LUMOs were calculated on the basis of their single-crystal structures at the cam-b3lyp/6-311+g(d,p) level. Stabilization energies were calculated by single-point calculations using the cam-b3lyp/6-311+g(d,p) method according to the equation *E* = *E*_complex_ – *E*_molecule_1_ – *E*_molecule_2_, where *E* is the stabilization energy, *E*_complex_ is the energy of the base pairs, *E*_molecule_1_ is the energy of molecule 1, and *E*_molecule_2_ is the energy of molecule 2. All of the electronic structure calculations were performed using Gaussian 09.

### NMR experiments

NMR experiments were performed using Bruker 600 MHz and 800 MHz NMR spectrometers (University of Florida, AMRIS). Samples were prepared as 150 µl solutions of DNA (2 mM) in 90% H_2_O + 10% D_2_O, or pure D_2_O, and included a phosphate buffer (50 mM) at pH 8.5 or 7.0; the solution was obtained by lyophilizing phosphate buffer at pH 8.5 or 7.0 and then resuspending it in pure D_2_O or H_2_O/D_2_O = 9:1 solution. Samples were analysed in a 5.0 mm/2.5 mm step-down tube at 25 °C.

For NOESY, COSY and TOCSY experiments in D_2_O, the spectral width was 4.2 kHz, the acquisition time was 243.8 ms, and the repetition delay was 2 s. The t1 delay (effective acquisition time delay), the evolution period during which nuclear spins interact and encode frequency information for the indirect dimension, was incremented to 60.7 ms (256 increments). The TOCSY experiments used MLEV-17 repetitions with mixing times of 15, 30 and 70 ms.

Two water-suppression methods were used in the NMR experiments using the 90% H_2_O + 10% D_2_O mix: pre-saturation water suppression and gradient (Watergate) water suppression. The amino group protons are likely to exchange with water; different water-suppression methods lead to various signal intensities.

### fZ-motif sensor system

The fluorescence of the DNA sample was visualized by fluorescence spectroscopy. ZZZ-FQ (1 µM) was dissolved in phosphate buffer (50 mM) solution (1 ml) in a quartz fluorescence cuvette. The pH of the buffer was cycled between 7 and 8.5 by alternatively adding 1 M HCl and 1 M NaOH at room temperature.

### Metal ion detection

ZZZ-FQ (1 µM) and various metal ions (5 mM) were dissolved in 50 mM phosphate buffer at 25 °C and then incubated overnight. The photographs of the different metal ions were taken using a Gel-image box under ultraviolet light (365 nm). The fluorescence intensities of every sample were measured using a Roche LightCycler 480. The excitation and emission filters were 490/10 and 520/10 nm.

## Online content

Any methods, additional references, Nature Portfolio reporting summaries, source data, extended data, supplementary information, acknowledgements, peer review information; details of author contributions and competing interests; and statements of data and code availability are available at 10.1038/s41557-024-01552-7.

## Supplementary information

Supplementary Information Supplementary Figs. 1–17, Tables 1–5 and mass and HPLC spectra.

Supplementary Data Statistical source data for supplementary figures.

## Source data

Source Data Fig. 2 Statistical source data for Fig. 2 and full-length, unprocessed gels for Fig. 2d–f.

Source Data Fig. 4 Statistical source data for Fig. 4.

## Data Availability

All data generated or analysed during this study, including information on materials and methods, optimization studies, experimental protocols, DFT calculations, NMR spectra, HPLC spectra and mass spectrometry, can be found within the main text of the article or its Supplementary Information. Furthermore, uncropped gel images and fluorescence curve data are available in the Source Data. Source data are provided with this paper.

## Code availability

This study did not utilize any custom code. The DFT calculations were performed using the Gaussian 09 software.
