# Supplementary material for: A folding motif formed with an expanded genetic alphabet
Source: Nat Chem. 2024 Jun 10;16(10):1715–22. doi: 10.1038/s41557-024-01552-7 (PMC11446821; doi:10.1038/s41557-024-01552-7)
Supplement: Supplementary file 1 — Supplementary Figs. 1–17, Tables 1–5 and mass and HPLC spectra. [file 41557_2024_1552_MOESM1_ESM.pdf]

nature chemistry

Supplementary information

https://doi.org/10.1038/s41557-024-01552-7

A folding motif formed with an expanded genetic alphabet

In the format provided by the authors and unedited

Table of Contents

Equipment and Material 2

Oligonucleotide synthesis (Supplementary Table 1) 3

Quinaldine Red for detect fZ-motif (Supplementary Figure S1) 4

C rich oligonucleotide melting curves (Supplementary Figure S2) 5

Z rich oligonucleotide melting curves (Supplementary Figure S3) 6

fZ-motif concentration independent melting temperature (warm) (Supplementary Figure S4) 7

fZ-motif concentration independent melting temperature (cool down) (Supplementary Figure S5) . 8

Various concentration of Magnesium ion affects the fZ-motif melting temperature (Supplementary Figure 6) 9

Various concentration of NaCl affects the fZ-motif melting temperature (Supplementary Figure 7) 10

Density functional theory calculations of Z base pair (Supplementary Figure 8; 9) (Table Supplementary 2) 11

Density functional theory calculations of C base pari (Supplementary Figure 10; 11; 12) (Supplementary Table 3) 12

The proton spectrum assignment of ZZZ in the pH=8.5 (Supplementary Table 4) 14

The proton spectrum assignment of ZZZ in the pH=7.0 at D_2O (Supplementary Table 5) 15

H-H COSY of ZZZ in the pH=8.5 at D_2O solution (Supplementary Figure 13) 16

The TOCSY spectrum of ZZZ in the pH=8.5 at D_2O solution (mixing time 15s) (Supplementary Figure 14) 17

The TOCSY spectrum of ZZZ in the pH=8.5 at D_2O solution (mixing time 30s) (Supplementary Figure 15) 18

The TOCSY spectrum of ZZZ in the pH=8.5 at D_2O solution (mixing time 70s) (Supplementary Figure 16) 19

The NOESY spectrum of ZZZ in the pH=8.5 at D_2O solution (Supplementary Figure 17) 20

ZZZ Mass spectrum and HPLC spectrum 21

Z-control 1 Mass spectrum and HPLC spectrum 22

Z-control 2 Mass spectrum and HPLC spectrum 23

Z-control 3 Mass spectrum and HPLC spectrum 24

ZZZ-FQ Mass spectrum and HPLC spectrum 25

ZZZ-control 4-FQ Mass spectrum and HPLC spectrum 26

Proton spectrum of dZ in the pH=6.0 at D_2O solution 27

Proton spectrum of dZ in the pH=7.0 at D_2O solution 28

Proton spectrum of dZ in the pH=8.5 at D_2O solution 28

Proton spectrum of dZ in the pH=9.0 at D_2O solution 28

References 29

Material:

Entry	Material	Company
1	Z-containing oligonucleotides	Firebird Biomolecular Sciences LLC (FL, US)
2	Nature base containing oligonucleotides	IDT (US)
3	Thioflavin T(THT)	Sigma–Aldrich (MO, USA)
4	Quinaldine red	Sigma–Aldrich (MO, USA)
5	Stains All	Sigma–Aldrich (MO, USA)
6	All metal salts	Sigma–Aldrich (MO, USA)
7	pH buffers	Home-made
8	Gel loading buffer (10X)	Thermo Fisher Scientific
9	dZ phosphoramidites	Firebird Biomolecular Sciences LLC (FL, US)
10	dZ nucleoside	Firebird Biomolecular Sciences LLC (FL, US)
11	FAM phosphoramidite	Glen Research
12	Dabcyl CPG	Glen Research
13	PAGE	Home-made

Equipment:

Entry	Equipment	Company
1	Microplate reader	Biotek Synergy
2	DNA synthesizer	ABI 394
3	PMI Scan	PMI
4	qPCR (LightCycler 480)	Roche
5	Gel-image box	Synoptics
6	Circular dichroism spectrometers	Chirascan
7	NMR	Bruker
8	Fluorescence spectroscopy	Agilent
9	Nanodrop 2000	Thermo Fisher Scientific

Oligonucleotide

Z-containing oligonucleotides were synthesized by Firebird Biomolecular Sciences LLC (FL, US) on an ABI 394 DNA synthesizer and purified by IE-HPLC. Natural oligonucleotides were from Integrated DNA Technologies (US). The concentrations of **Z**-containing DNA were quantified using 280 um UV absorbance with a Nanodrop 2000 UV spectrophotometer.

Supplementary Table 1. DNA synthesized by Firebird Biomolecular Sciences LLC ("Firebird").

Name	Sequence (5’ to 3’)	Source	Target Masses	Observed Masses
ZZZ	AZZZTAAZZZTAAZZZTAAZZZT	Firebird	7345.6	7344.0
Z-control 1	AZTZAZAZAZZAZZTZTZAZTZT	Firebird	7336.6	7335.7
Z-control 2	AZZZTAATTTTAATTTTAAZZZT	Firebird	7171.6	7170.9
Z-control 3	AZZZTAATTTTAAZZZTAAZZZT	Firebird	7258.6	7257.8
ZZZ-FQ	FAM-AZZZTAAZZZTAAZZZTAAZZZT-dabcyl	Firebird	8345.3	8345.7
ZZZ-control 4-FQ	FAM-AZZZTAACCCTAAZZZTAACCCT-dabcyl	Firebird	8081.3	8082.3

TEST	DESCRIPTION	METHOD	RESULT
**MASS**	The relative error of the molecular weights ≤0.2%	ESI	Pass
**HPLC**	IE-HPLC analysis purity >90%	IE-HPLC	Pass

Quinaldine Red for detect fZ-motif

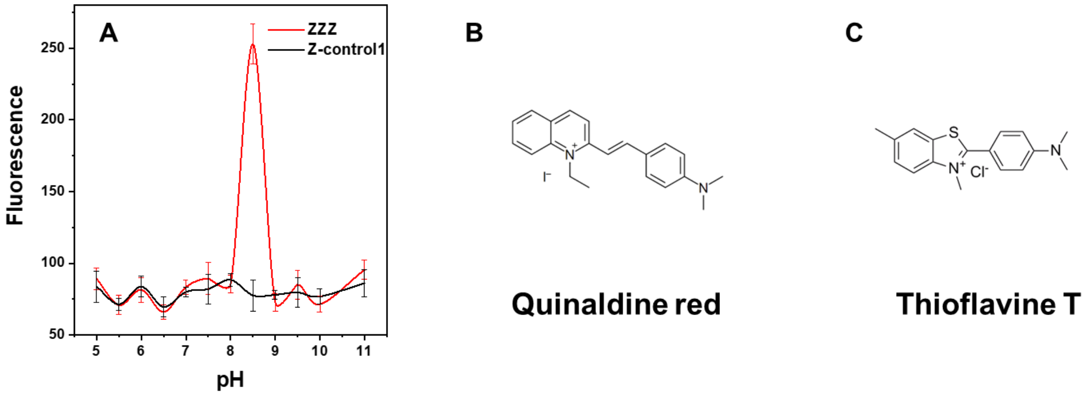

Supplementary Figure 1. Detection of fZ-Motif Formation Using Quinaldine Red Across Different pH Conditions. (**A red**) Intensity of the fluorescence arising from the intercalation of quinaldine red into the **Z**-rich sequence (ZZZ). (**A black**) Substantially less quinaldine red fluorescence is observed with the randomized Z-control 1 oligonucleotide. All samples contained 1.0 μM DNA and 6.0 μM quinaldine red in 100 mM PB buffer with various pH at 25 ℃. Fluorescence data were collected using Greiner Bio-One 96-well micro-plates read by a Biotek Synergy 2 microplate reader. Excitation and emission filters were 550/15 and 630/15 nm. n = 5 independent experiments. Error bars in (a) represent mean values ± s.d.

(B) The chemical structure of quinaldine red. (C) The chemical structure of Thioflavine T.

C rich oligonucleotide melting curves

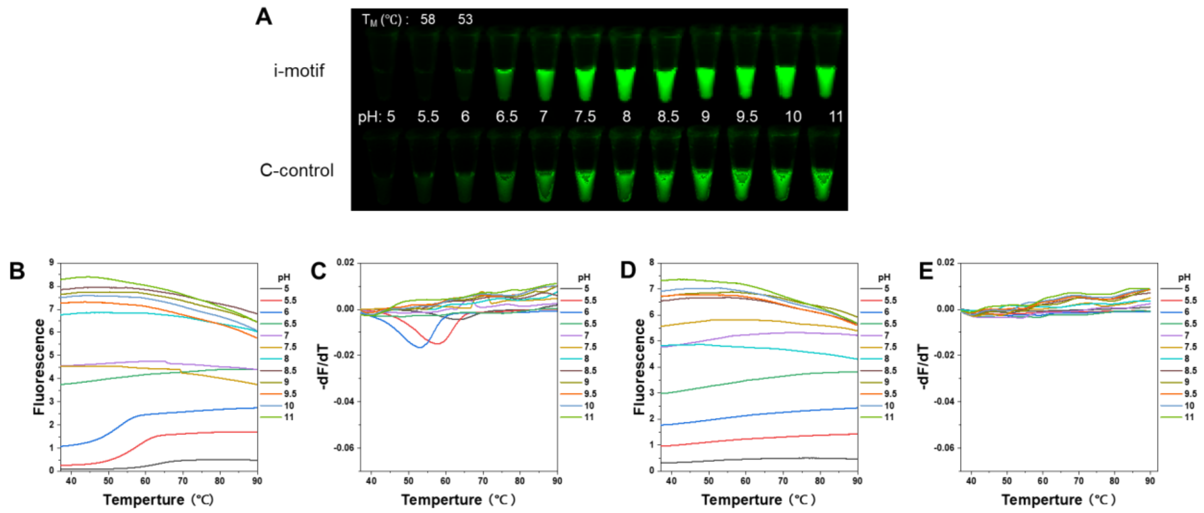

Supplementary Figure 2. Melting curves showing the pH and temperature dependence of FAM fluorescence in C-rich oligonucleotides. (A) Photographs of the fluorescence of DNA samples (1 μM) as a function of pH from 5.0 to 11 at 25 ℃. (B) The T_m curve of i-moitf-FQ by recording the fluorescence of FAM from pH 5.0 to 11 in a Roche LightCycler 480 by warming from 37℃ to 90 ℃ with a melting curve setting (5 ℃/min), (C) Melting peaks of i-motif-FQ plotted by -dF/dT vs. temperature. (D) The T_m curve of C-control-FQ by recording the fluorescence of FAM from pH 5.0 to 11 in a Roche LightCycler 480 by warming from 37℃ to 90 ℃ with a melting curve setting (5 ℃/min). (E) Melting peaks of C-control-FQ plotted by -dF/dT vs. temperature. Note that FAM loses its fluorescence below pH 6.5 by protonation of the chromophore, which leads to ring closure.

Z rich oligonucleotide melting curves

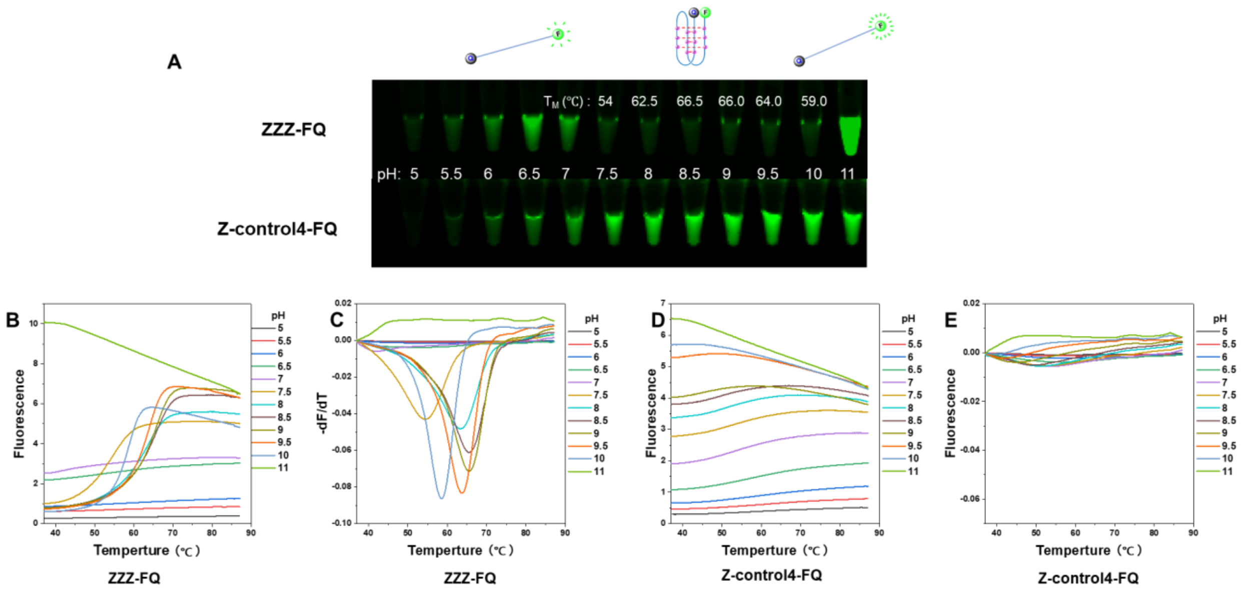

Supplementary Figure 3. Melting curves showing the pH and temperature dependence of FAM fluorescence in Z-rich oligonucleotides. (A) Photographs of the fluorescence of DNA samples (1 µM) at pH values from pH 5.0 to 11 at 25 ℃. (B) The T_m curve of ZZZ-FQ by recording the fluorescence of FAM at pHs from 5.0 to 11 in a Roche LightCycler 480 by warming from 37℃ to 90 ℃ with a melting curve setting (5 ℃/min), (C) Melting peaks of ZZZ-FQ plotted by -dF/dT versus temperature. (D) The T_m curve of Z-control4-FQ by recording fluorescence of FAM from pH 5.0 to 11 in a Roche LightCycler 480 by warming from 37℃ to 90 ℃ with a melting curve setting (5 ℃/min). (E) Melting peaks of Z-control4-FQ plotted by -dF/dT versus temperature. Note that FAM loses its fluorescence below pH 6.5.

fZ-motif Concentration independent melting temperature (warm curve)

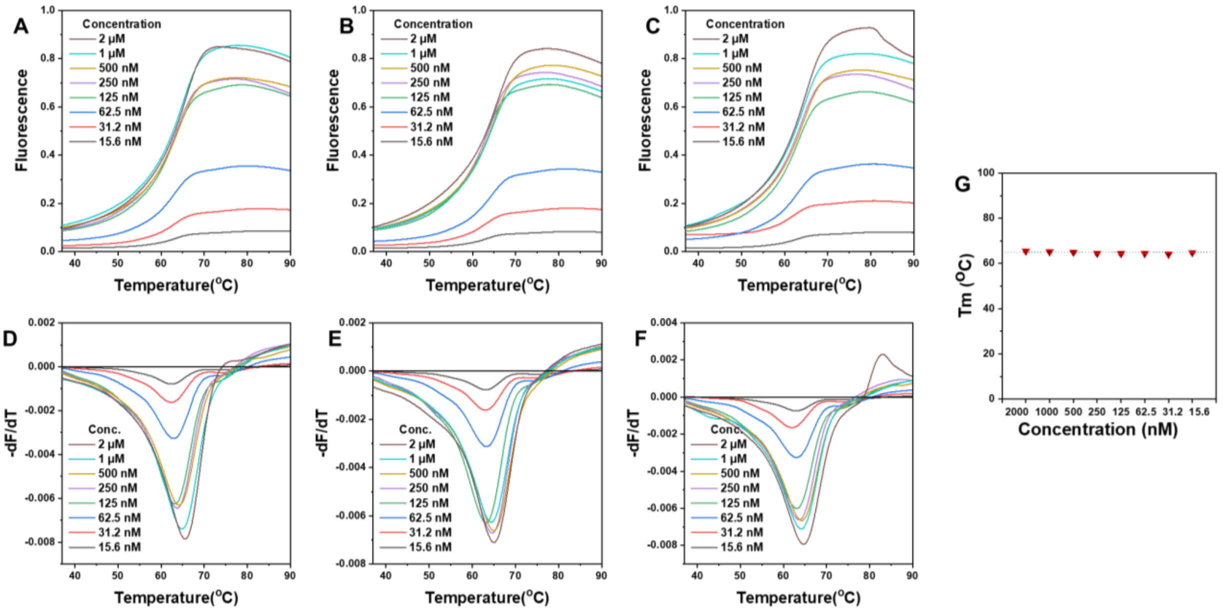

Supplementary Figure 4. Melting curves showing the concentration independence of FAM fluorescence in Z-rich oligonucleotides at pH 8.5 in PB buffer. (A, B, C) The T_m curve of ZZZ-FQ by recording the fluorescence of FAM with concentration from 2 μM to 15 nM in a Roche LightCycler 480 by warming from 37℃ to 90 ℃ with a melting curve setting (5 ℃/min). A; B; C are three parallel experiments. (D, E, F) Melting peaks of ZZZ-FQ plotted by -dF/dT versus temperature of various DNA concentration. D; E; F are three parallel experiments corresponding to A; B; C. (G) The T_m values of ZZZ-FQ in various concentration. n = 3 independent experiments. Error bars in (G) represent mean values ± s.d.

Entry	1	2	3	4	5	6	7	8
DNA Conc.	2 μM	1 μM	500 nM	250 nM	125 nM	62.5 nM	31.2 nM	15.6 nM
Volume	5 μl	10 μl	20 μl	40 μl	80 μl	80 μl	80 μl	80 μl

fZ-motif Concentration independent melting temperature (Cool down curve)

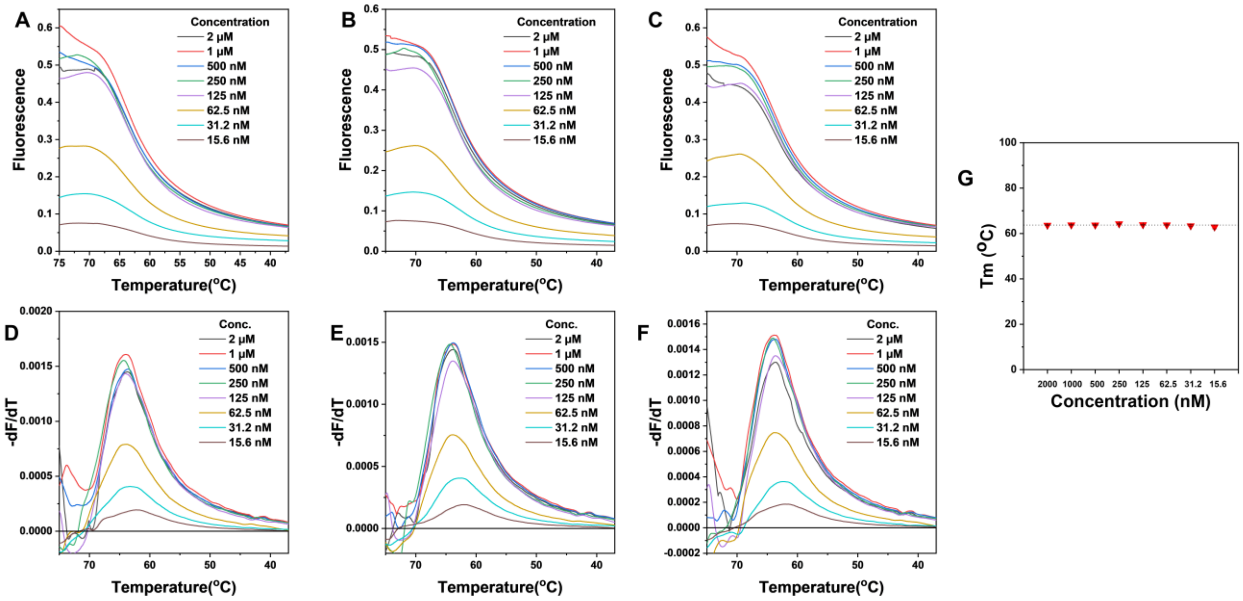

Supplementary Figure 5. Melting curves showing the concentration independence of FAM fluorescence in Z-rich oligonucleotides at pH 8.5 in PB buffer. (A, B, C) The T_m curve of ZZZ-FQ by recording the fluorescence of FAM with concentration from 2 μM to 15 nM in a Roche LightCycler 480 by cooling down from 75℃ to 37 ℃ with a melting curve setting (0.04 ℃/s). A; B; C are three parallel experiments. (D, E, F) Melting peaks of ZZZ-FQ plotted by -dF/dT versus temperature of various DNA concentration. D; E; F are three parallel experiments corresponding to A; B; C. (G) The T_m values of ZZZ-FQ in various concentration. n = 3 independent experiments. Error bars in (G) represent mean values ± s.d.

Entry	1	2	3	4	5	6	7	8
DNA Conc.	2 μM	1 μM	500 nM	250 nM	125 nM	62.5 nM	31.2 nM	15.6 nM
Volume	5 μl	10 μl	20 μl	40 μl	80 μl	80 μl	80 μl	80 μl

Various concentration of Magnesium ion affects the fZ-motif melting temperature

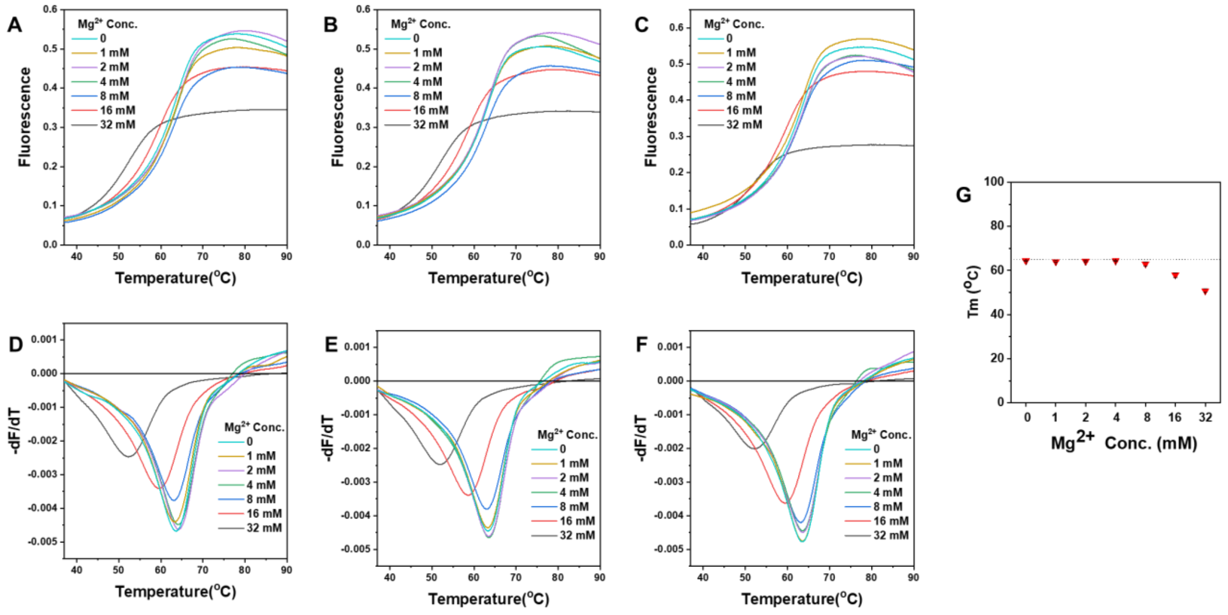

Supplementary Figure 6. Melting curves showing the additional magnesium ion (Mg^{2+}) concentration independence of FAM fluorescence in Z-rich oligonucleotides (1 μM) at pH 8.5 in PB buffer. (A, B, C) The T_m curve of ZZZ-FQ by recording the fluorescence of FAM with additional magnesium ion (Mg^{2+}) concentration from 0 to 32 mM in a Roche LightCycler 480 by warming from 37℃ to 90 ℃ with a melting curve setting (5 ℃/min). A; B; C are three parallel experiments. (D, E, F) Melting peaks of ZZZ-FQ plotted by -dF/dT versus temperature of various additional magnesium ion (Mg^{2+}) concentration. D; E; F are three parallel experiments corresponding to A; B; C. (G) The T_m values of ZZZ-FQ under various additional magnesium ion (Mg^{2+}) concentration. n = 3 independent experiments. Error bars in (G) represent mean values ± s.d. Notes: precipitates were seen with the condition of high concentration of Mg^{2+} (16 mM and 32 mM).

Entry	1	2	3	4	5	6	7
Additional (Mg^{2+}) Concentration	0 mM	1 mM	2 mM	4 mM	8 mM	16 mM	32 mM

Various concentration of NaCl affects the fZ-motif melting temperature

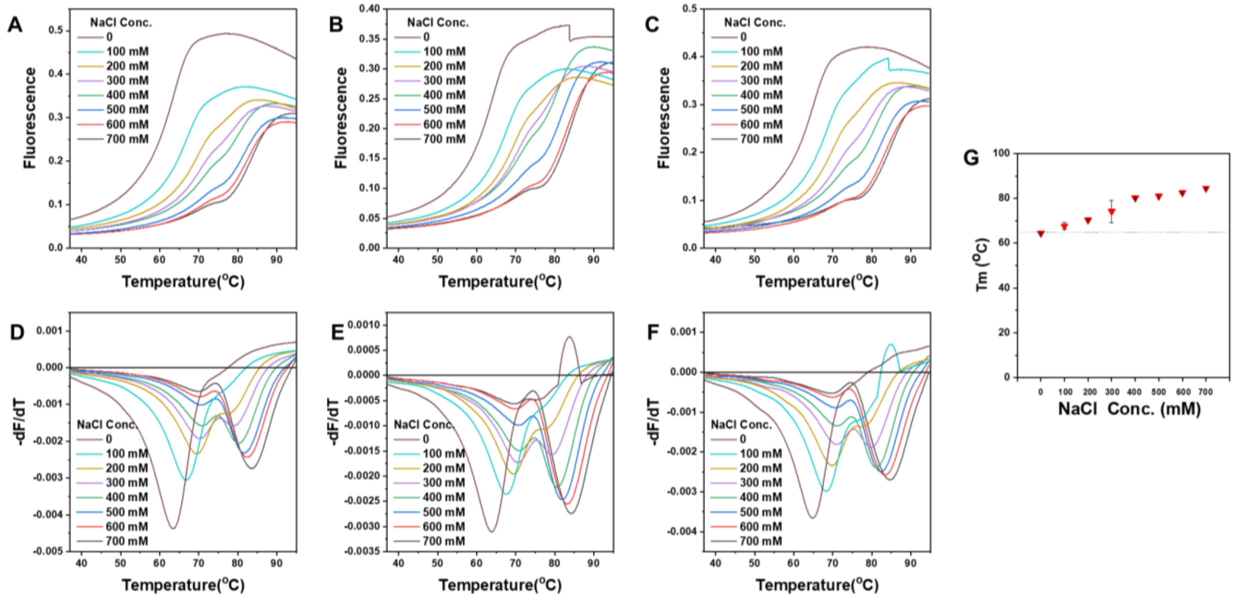

Supplementary Figure 7. Melting curves showing the additional various concentration of NaCl affects the FAM fluorescence in Z-rich oligonucleotides (1 μM) at pH 8.5 in PB buffer. (A, B, C) The T_m curve of ZZZ-FQ by recording the fluorescence of FAM with additional NaCl concentration from 100 to 800 mM in a Roche LightCycler 480 by warming from 37℃ to 95 ℃ with a melting curve setting (5 ℃/min). A; B; C are three parallel experiments. (D, E, F) Melting peaks of ZZZ-FQ plotted by -dF/dT versus temperature of various additional NaCl concentration. D; E; F are three parallel experiments corresponding to A; B; C. (G) The T_m values of ZZZ-FQ under various additional NaCl concentration. n = 3 independent experiments. Error bars in (G) represent mean values ± s.d.

Entry	1	2	3	4	5	6	7	8
Additional (NaCl) Conc. (mM)	0	100	200	300	400	500	600	700

Theoretical calculations of Z base pair

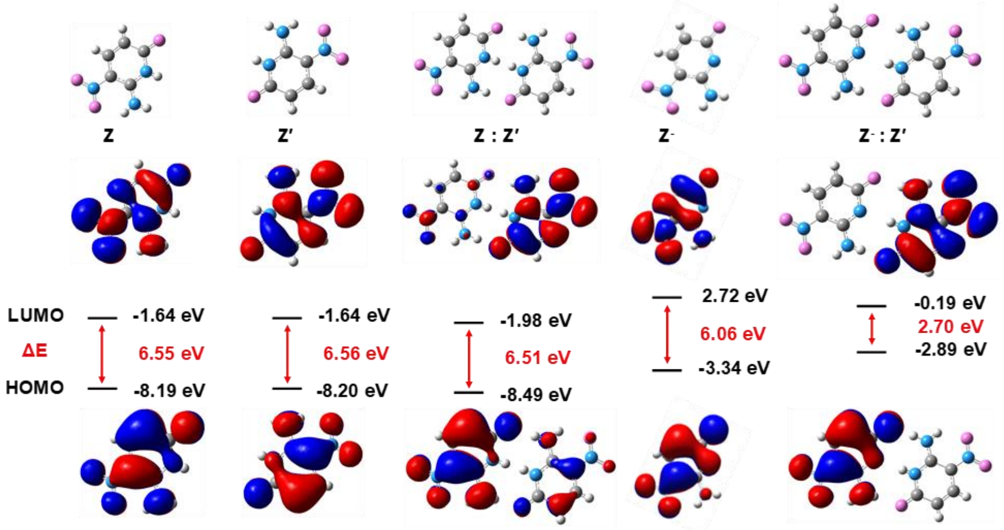

Supplementary Figure 8. Structure, molecular orbitals distributions, energy levels and energy gaps of HOMO and LUMO of **Z**, **Z′**, **Z**$^-$, **Z : Z′** and **Z**$^-$ **: Z′** base. (Z’ is same with Z, which in a different orientation. **Z**$^-$:**Z** pairing observed in the structure of the crystal formed from the aminonitropyridone heterocycle alone[1])

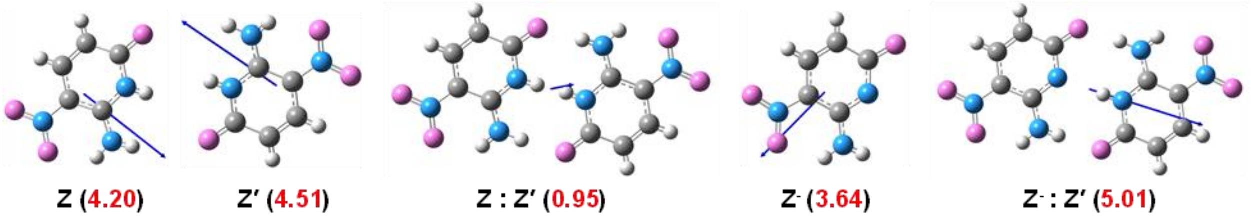

Supplementary Figure 9. The dipole moments (in debye) of **Z**, **Z′**, **Z**$^-$, **Z : Z′** and **Z**$^-$ **: Z′** base.

Supplementary Table 2. The calculated energy levels, energy gaps, total energy and diploe moment of **Z**, **Z′**, **Z**$^-$, **Z : Z′** and **Z**$^-$ **: Z′** base.

Entry	LUMO (eV)	HOMO (ev)	ΔE (eV)	E (kcal/mol)	Dipole moment (D)
**Z**	-1.64	-8.19	6.54	-365957.55	4.20
**Z′**	-1.64	-8.20	6.56	-365968.79	4.51
**Z : Z′**	-1.98	-8.49	6.51	-731928.27	0.95
**Z**$^-$	2.72	-3.34	6.06	-365651.94	3.64
**Z**$^-$ **: Z′**	-0.19	-2.89	2.70	-731967.19	5.01

Hydrogen bond stabilization energy of **Z : Z′** = $E_Z + E_{Z'} - E_{Z:Z'}$ (1.93 kcal/mol)
Hydrogen bond stabilization energy of **Z**$^-$ **: Z′** = $E_{Z^-} + E_{Z'} - E_{Z^-:Z'}$ (346.46 kcal/mol)

Theoretical calculations of C base pair

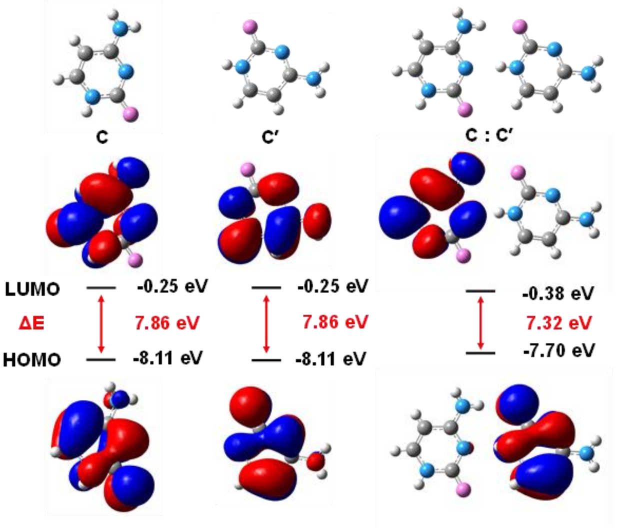

Supplementary Figure 10. Structure, molecular orbitals distributions, energy levels and energy gaps of HOMO and LUMO of **C**, **C′**, and **C : C′** base[2]. (C’ is same with C, which in a different orientation)

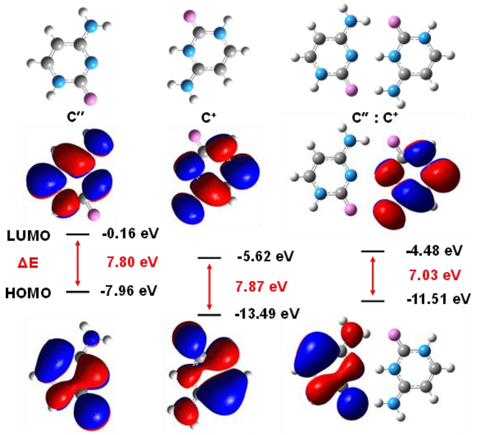

Supplementary Figure 11. Structure, molecular orbitals distributions, energy levels and energy gaps of HOMO and LUMO of **C″**, **C+**, and **C″ : C+** base[3]. (C’’ is normal C) CCDC: 664138.

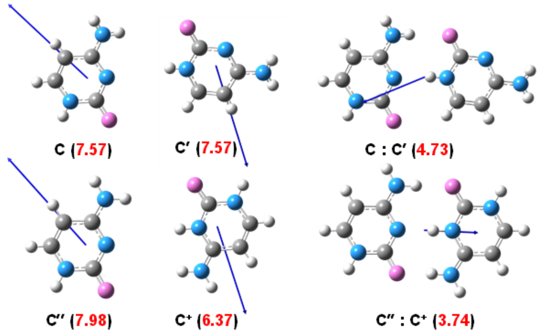

Supplementary Figure 12. The diploe moment direction of **C**, **C′**, **C : C′, C″**, **C+**, and **C″ : C+** base.

Supplementary Table 3. The calculated energy levels, energy gaps, total energy and diploe moment of **C**, **C′**, **C : C′, C″**, **C**$^{+}$, and **C″ : C**$^{+}$ base

Entry	LUMO (eV)	HOMO (ev)	ΔE (eV)	E (kcal/mol)	Dipole moment (D)
**C**	-0.25	-8.11	7.86	-247714.76	7.57
**C′**	-0.25	-8.11	7.86	-247714.72	7.57
**C : C′**	-0.38	-7.70	7.32	-495446.95	4.73
**C″**	-0.16	-7.96	7.80	-247775.59	7.98
**C**$^{+}$	-5.62	-13.49	7.87	-248010.28	6.37
**C″: C**$^{+}$	-4.48	-11.51	7.03	-495835.06	3.74

Hydrogen bond stabilization energy of **C : C′** = $E_{C} + E_{C'} - E_{C:C'}$ (17.47 kcal/mol)
Hydrogen bond stabilization energy of **C**$^{+}$ **: C″** = $E_{C''} + E_{C^{+}} - E_{C'':C^{+}}$ (49.19 kcal/mol)

The proton spectrum assignment of ZZZ in the pH=8.5

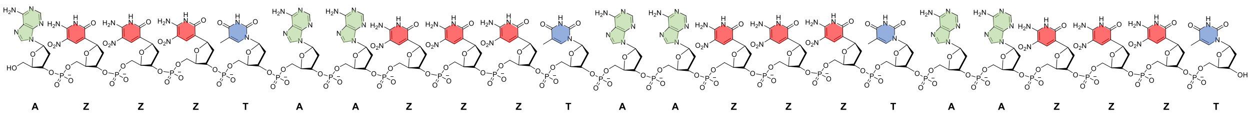

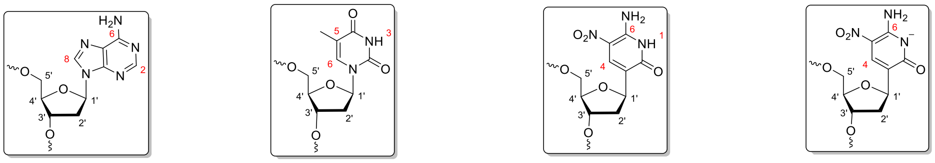

A T Z Z^-

Supplementary Table 4. The proton spectrum assignment of ZZZ at pH 8.5 at D_2O solution or at H_2O/D_2O (9:1). (The amino proton signal assignment from the proton spectrum at solution H_2O/D_2O (9:1)).

δ (ppm) / Base	1’	2’ (a/b)	3’	4’	5’ (a/b)	1	2	3	4	6	8
Z	6.07br	2.87br/2.57br	4.91br	5.64br	4.00m/3.90m	9.71t			6.90d	8.02s	
Z^-	5.25m	1.98m/1.84m	4.53m	4.63m	4.03m/3.91m				7.30d	7.71s, 7.38s	
T	6.03t	2.14m/2.09m	4.83br	4.38m	4.00m/3.89m			10.92s; 9.01s	1.47s; 1.51s; 1.47s; 1.60s.	7.29s; 7.32s; 7.29s; 7.47s.	
A	6.15t	2.76m/2.46br	4.17br	4.71m	3.63m/3.59m		7.95s				7.56s

The proton spectrum assignment of ZZZ in the pH=7.0 at D_2O

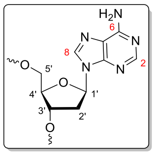

A

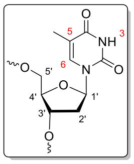

T

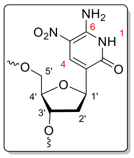

Z

Supplementary Table 5. The proton spectrum assignment of ZZZ in the pH=7.0 at D_2O solution.

(ppm)	1'	2' (a/b)	3'	4'	5' (a/b)	2	4	5	6	8
A	6.15t, 6.19t	2.75br; 2.65br; 2.22br	4.49br	4.01br	3.92m, 3.98m	8.02 m				7.56s
Z	6.02m	2.61br; 2.75br	4.92br	4.31br	4.05m		7.51m (8.09m)			
T	5.84m	2.41br; 2.52br	4.83br	4.21br; 4.29br	3.83m, 3.92m			1.71s; 1.67s; 1.65s;	7.53s; 7.21s; 7.27s; 7.23s	

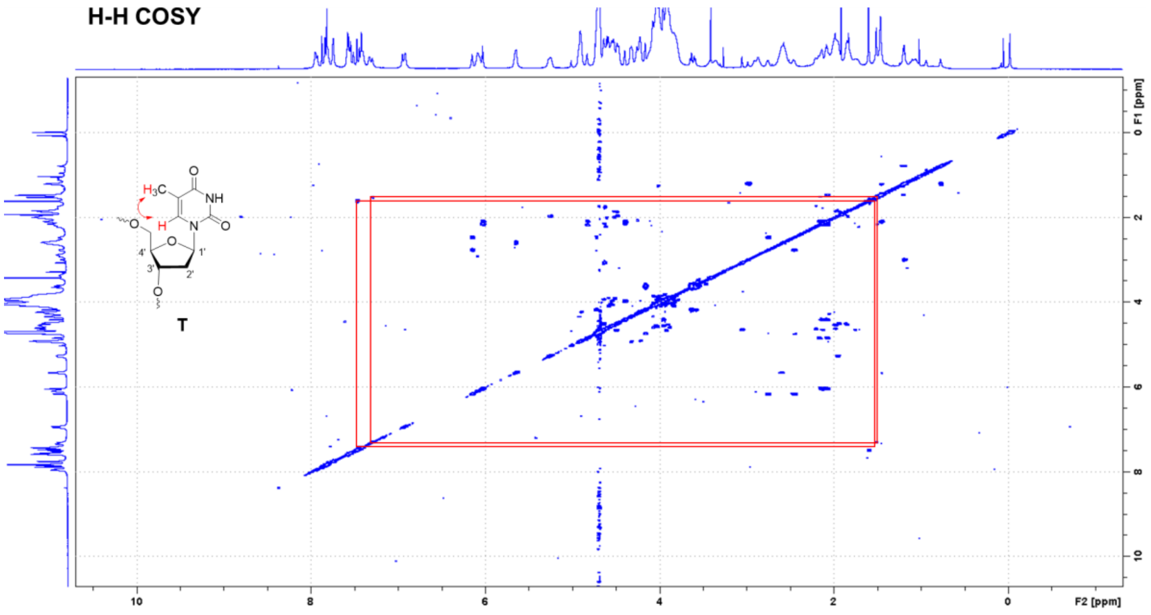

Supplementary Figure 13. The COSY spectrum of ZZZ at pH=8.5 in D_2O highlighting cross-peaks observed between three CH_3-protons in the three thymidines (1.47, 1.47, and 1.51 ppm) and the corresponding 6-position protons (7.3 to 7.5 ppm).

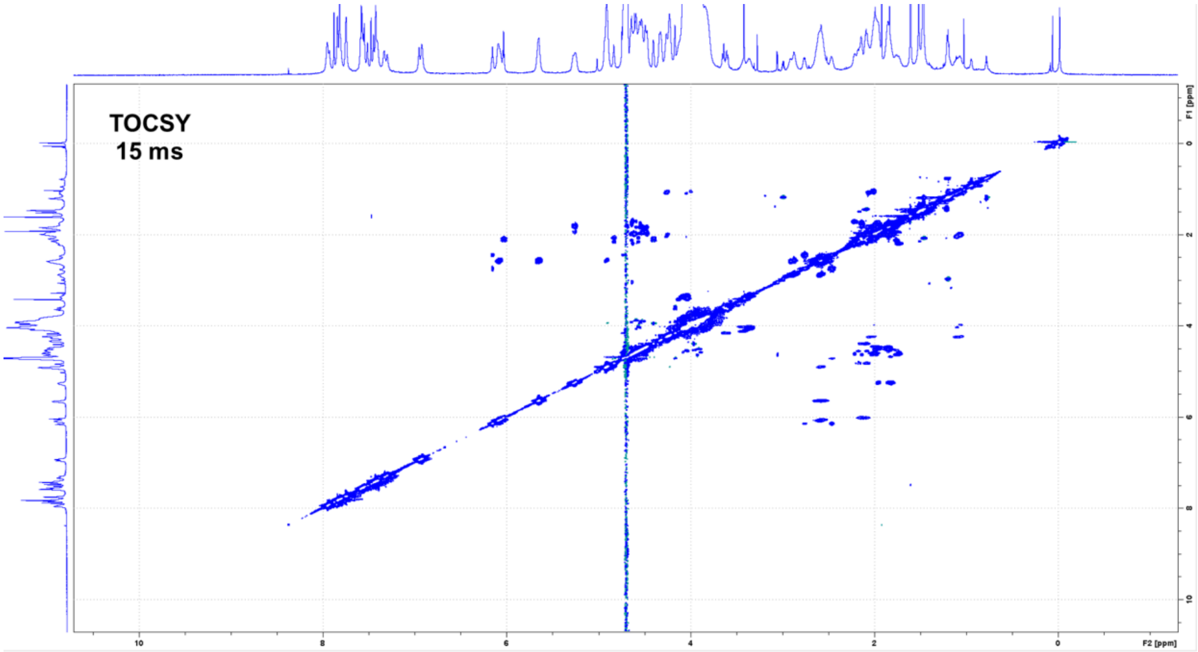

Supplementary Figure 14. The TOCSY spectrum of ZZZ in buffered D_2O solution. MLEV-17 repetitions were used with mixing times of 15 ms.

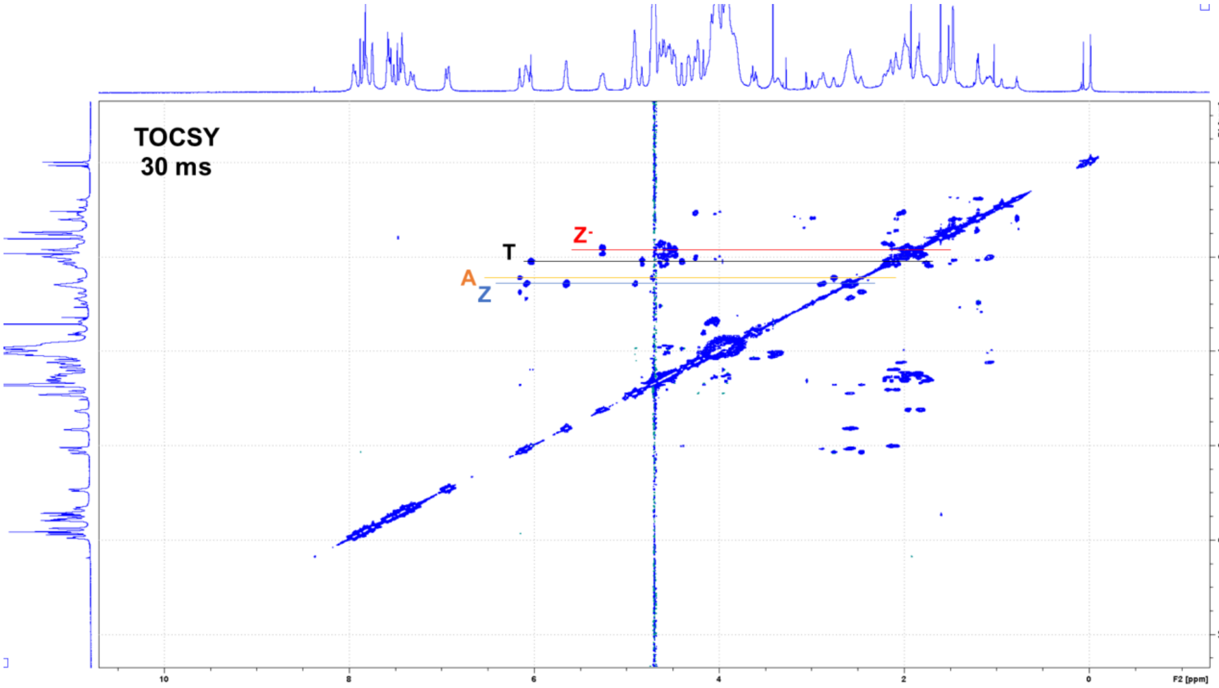

Supplementary Figure 15. The TOCSY spectrum of ZZZ at pH=8.5 in D_2O, using MLEV-17 repetitions with mixing times of 30 ms. Indicated by lines are cross peaks between sugar ring protons in the indicated spin systems. Characteristic TOCSY fingerprint regions show sugar ring proton connectivity in the A, T, Z, and Z^- spin systems.

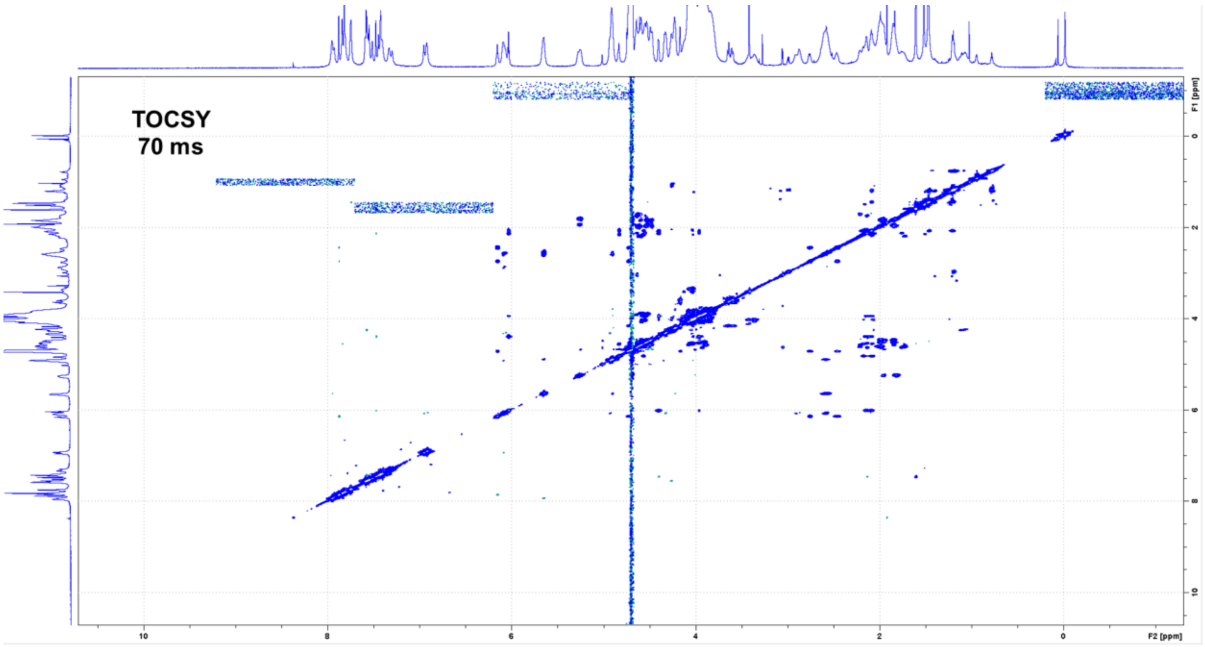

Supplementary Figure 16. The TOCSY spectrum of ZZZ at pH 8.5 in D_2O using MLEV-17 repetitions with mixing times of 70 ms.

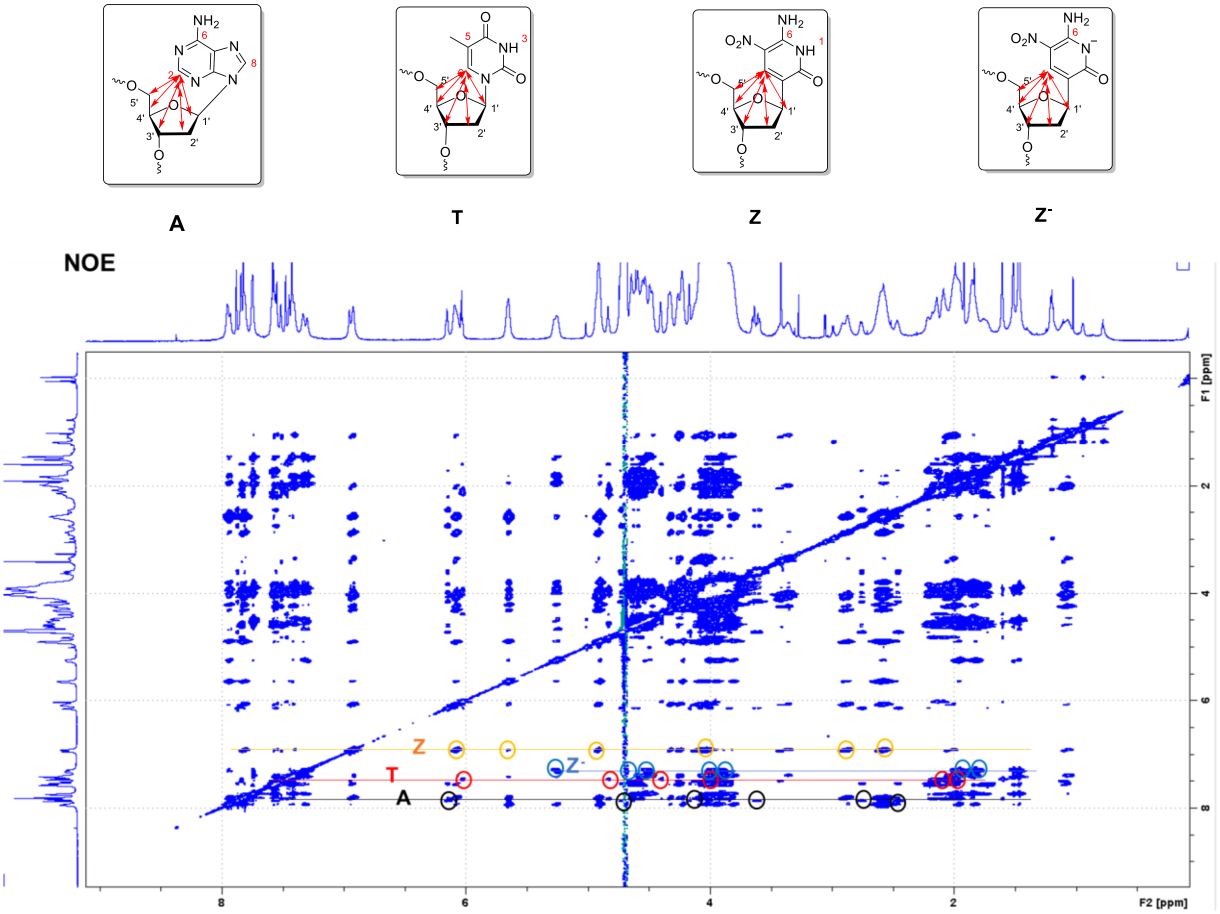

Supplementary Figure 17. The NOESY spectrum of ZZZ at pH 8.5 in D_2O. Characteristic NOE fingerprints show the A, T, Z, and Z^- sugar ring proton connectivity. NOESY correlation of the proton in nucleoside with sugar protons were indicated by lines with different colors for the A, T, Z, and Z^- spin systems. Notice the upfield shift of C1' when Z is deprotonated.

ZZZ: Target Masses: 7345.6 Observed Masses:7344.0

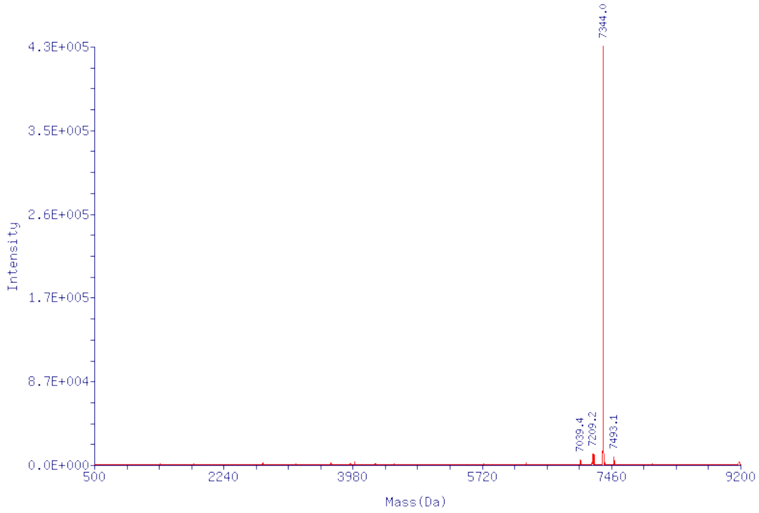

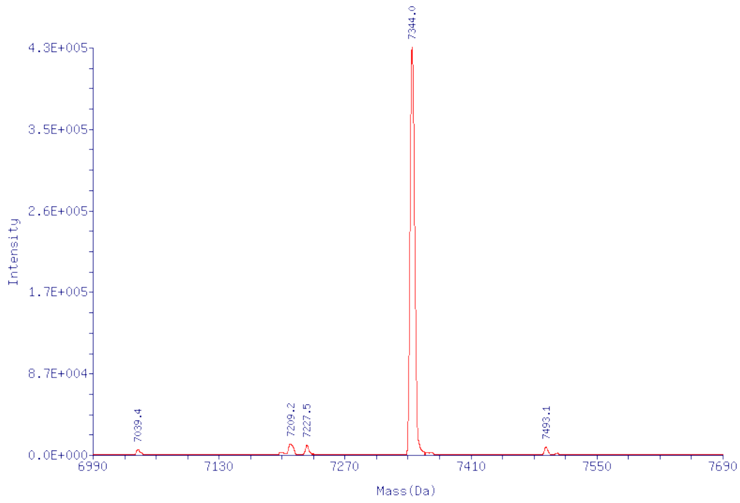

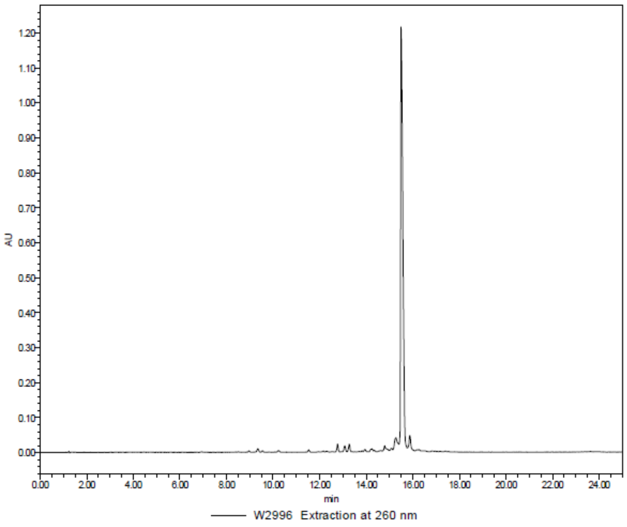

(HPLC)

Z-control 1: Target Masses: 7336.6 Observed Masses:7335.7

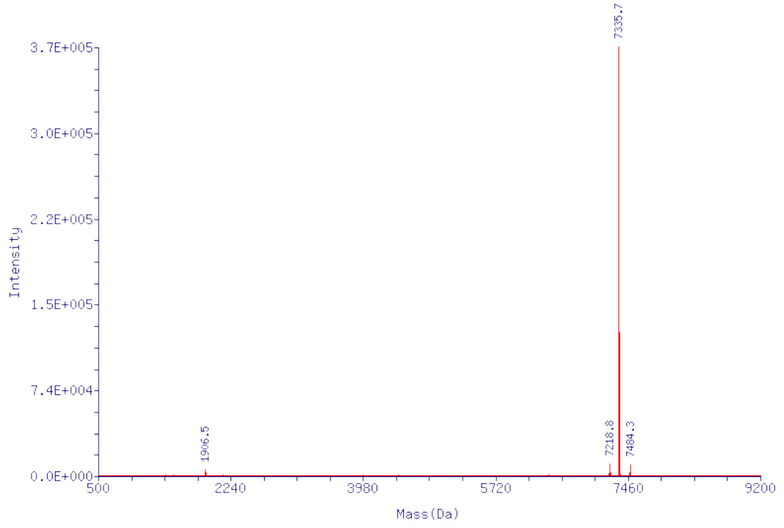

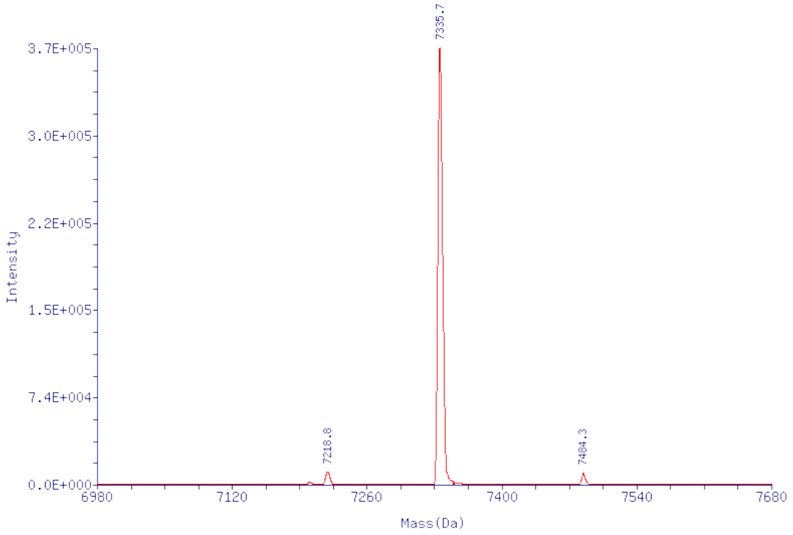

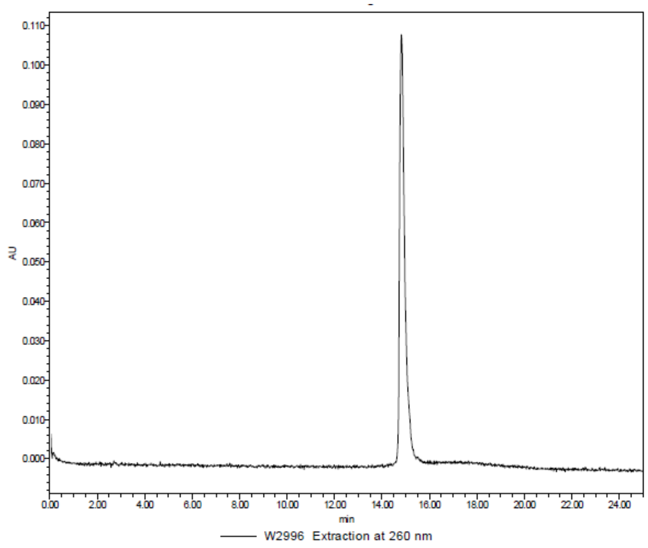

(HPLC)

Z-control 2: Target Masses: 7171.6 Observed Masses:7170.9

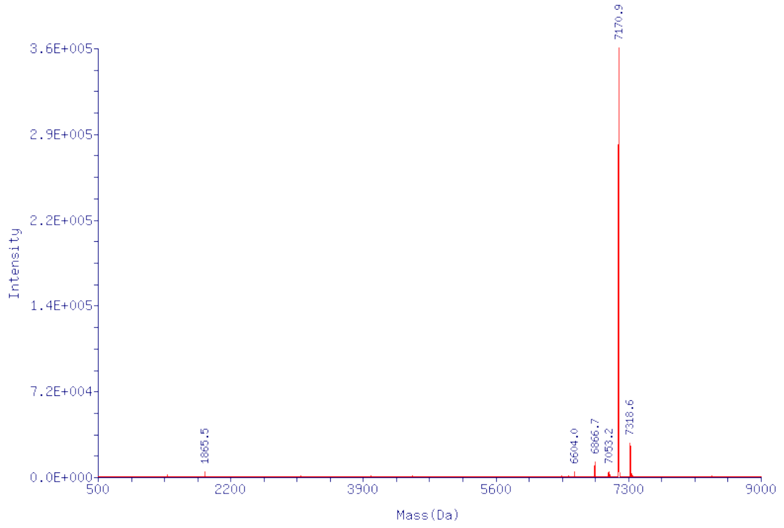

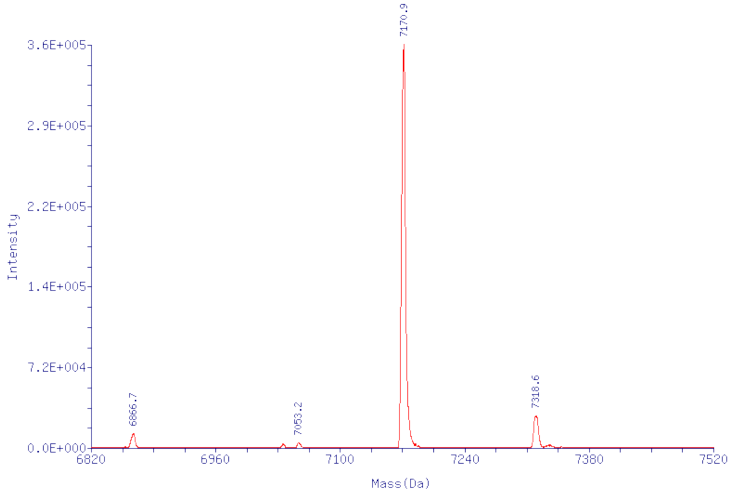

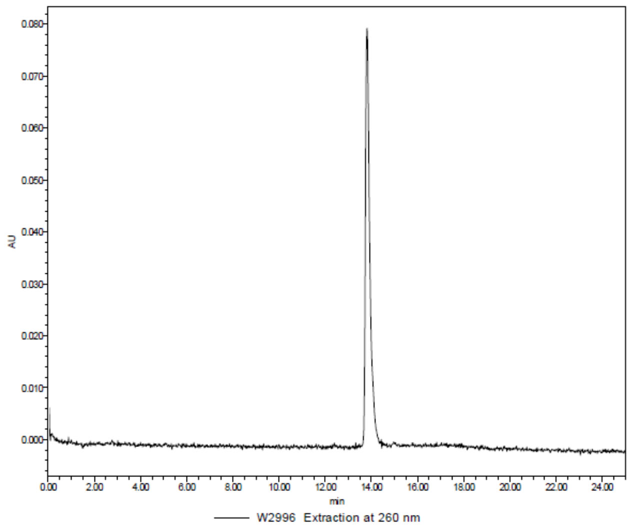

(HPLC)

Z-control 3: Target Masses: 7258.6   Observed Masses:7257.8

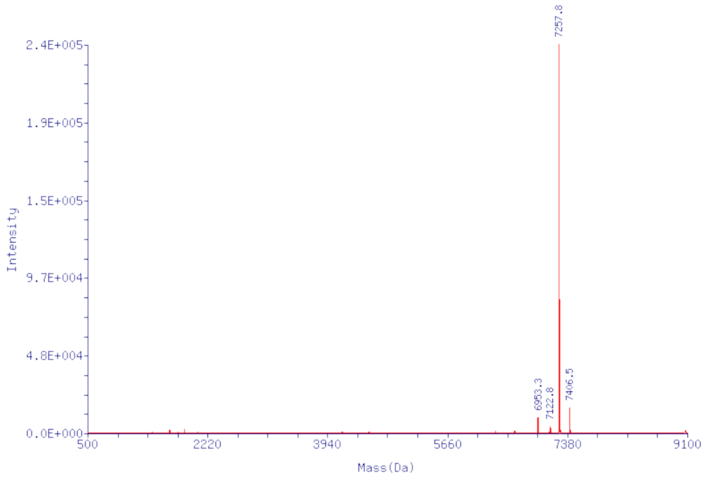

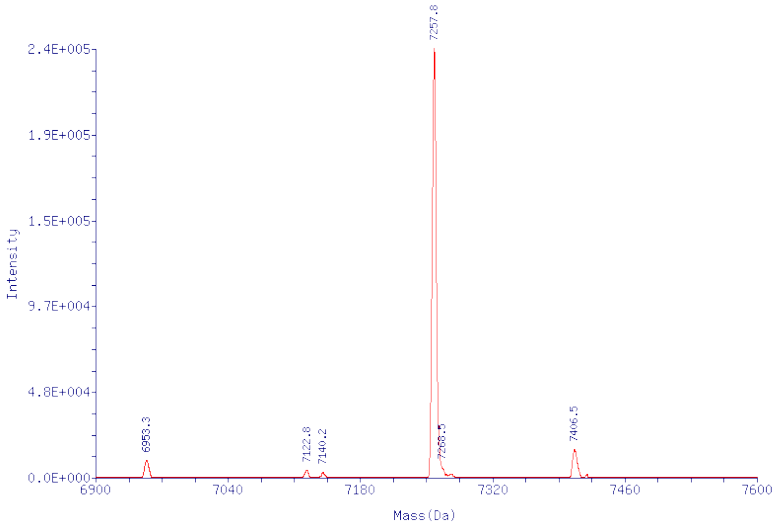

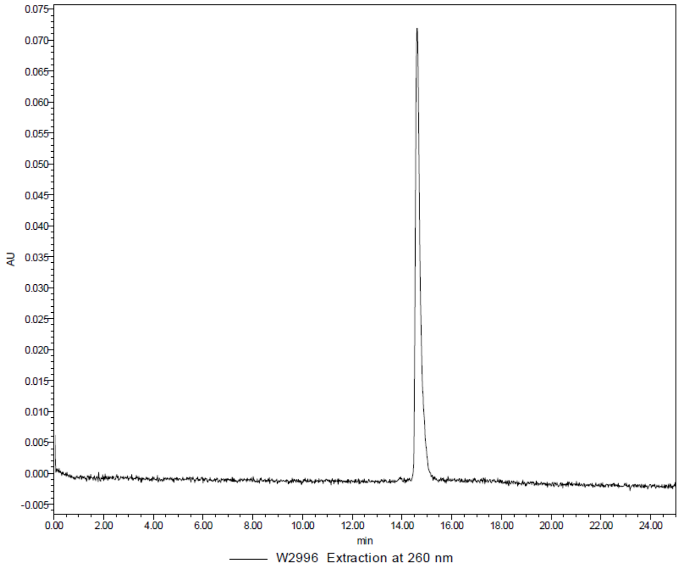

(HPLC)

ZZZ-FQ, Target Masses: 8345.3, Observed Masses: 8345.7

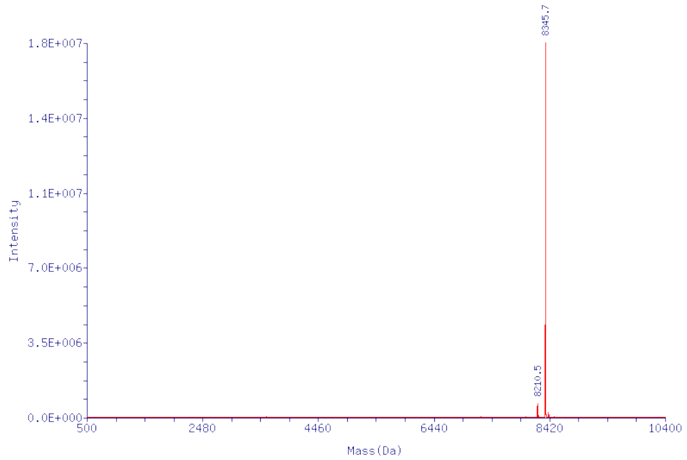

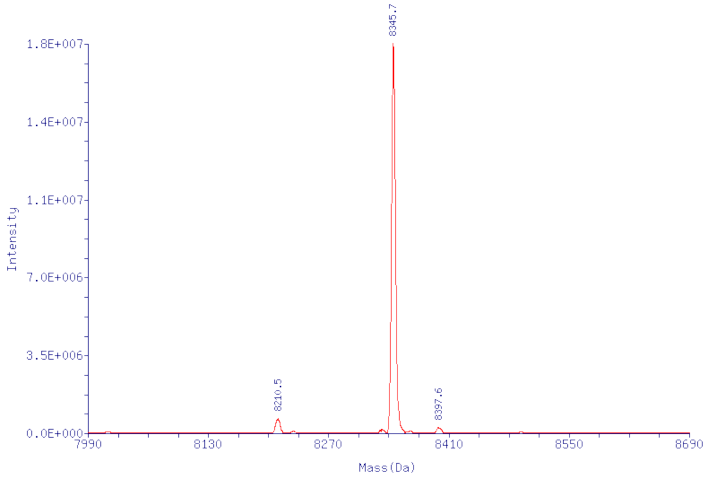

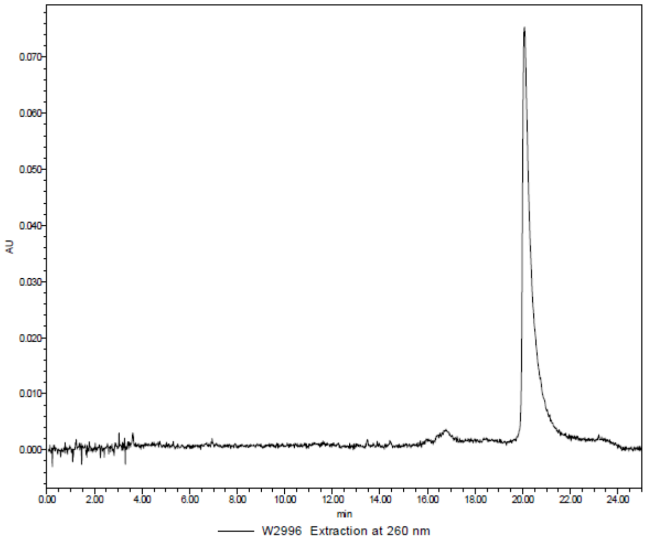

(HPLC)

Z-control 4-FQ: Target Masses: 8081.3   Observed Masses: 8082.3

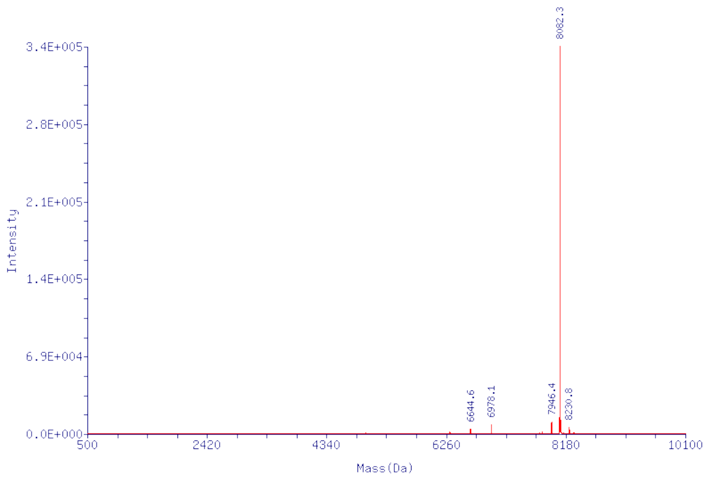

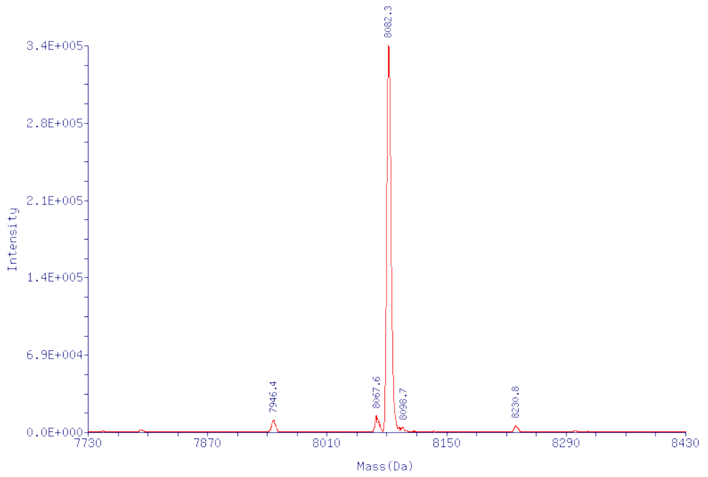

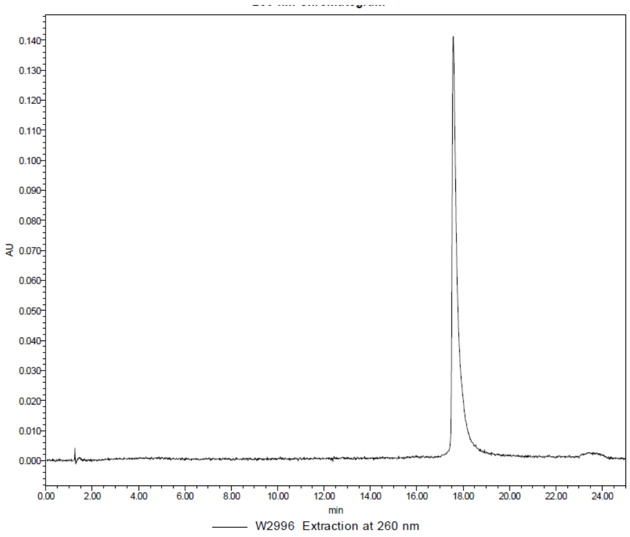

(HPLC)

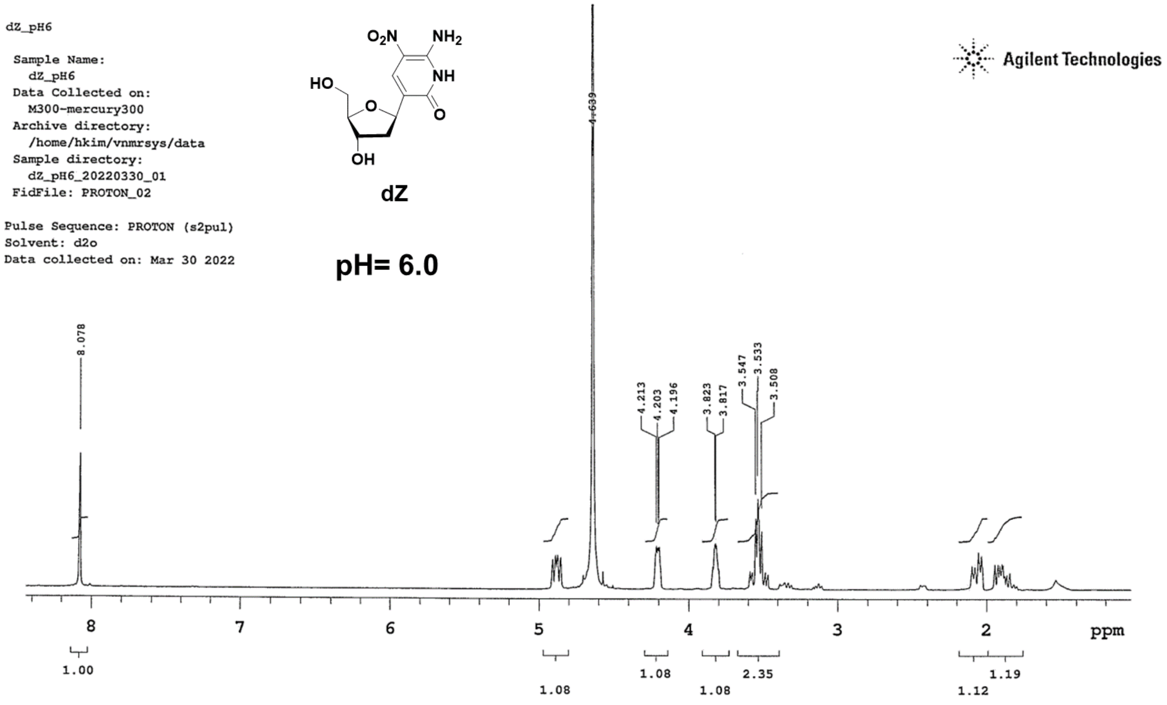
dZ_pH6
Sample Name:
dZ_pH6
Data Collected on:
M300-mercury300
Archive directory:
/home/hkim/vnmrsys/data
Sample directory:
dZ_pH6_20220330_01
FidFile: PROTON_02
Pulse Sequence: PROTON (s2pul)
Solvent: d2o
Data collected on: Mar 30 2022
O2N
NH2
NH
HO
O
O
OH
dZ
pH= 6.0
Agilent Technologies
8.078
4.639
4.213
4.203
4.196
3.823
3.817
3.547
3.533
3.508
8
7
6
5
4
3
2
ppm
1.00
1.08
1.08
1.08
2.35
1.12
1.19

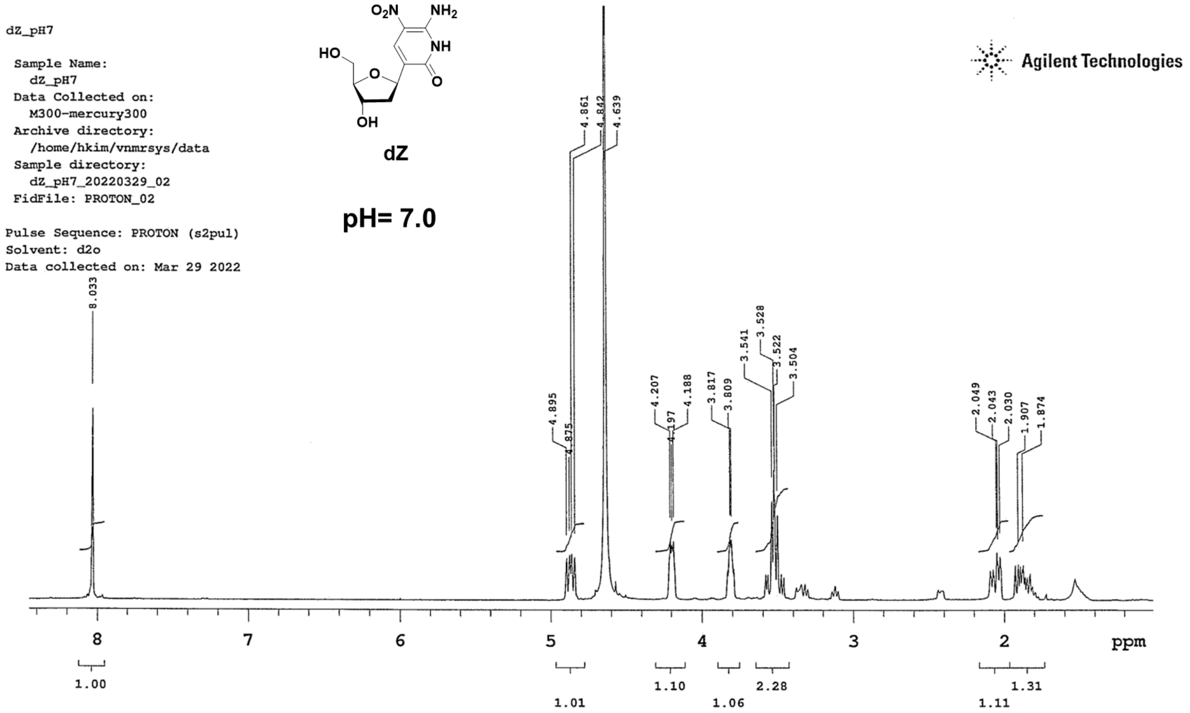
dZ_pH7
Sample Name:
dZ_pH7
Data Collected on:
M300-mercury300
Archive directory:
/home/hkim/vnmrsys/data
Sample directory:
dZ_pH7_20220329_02
FidFile: PROTON_02
Pulse Sequence: PROTON (s2pul)
Solvent: d2o
Data collected on: Mar 29 2022
O2N
NH2
NH
HO
O
O
OH
dZ
pH= 7.0
Agilent Technologies
8.033
4.895
4.875
4.861
4.842
4.639
4.207
4.197
4.188
3.817
3.809
3.541
3.528
3.522
3.504
2.049
2.043
2.030
1.907
1.874
8
7
6
5
4
3
2
ppm
1.00
1.01
1.10
1.06
2.28
1.11
1.31

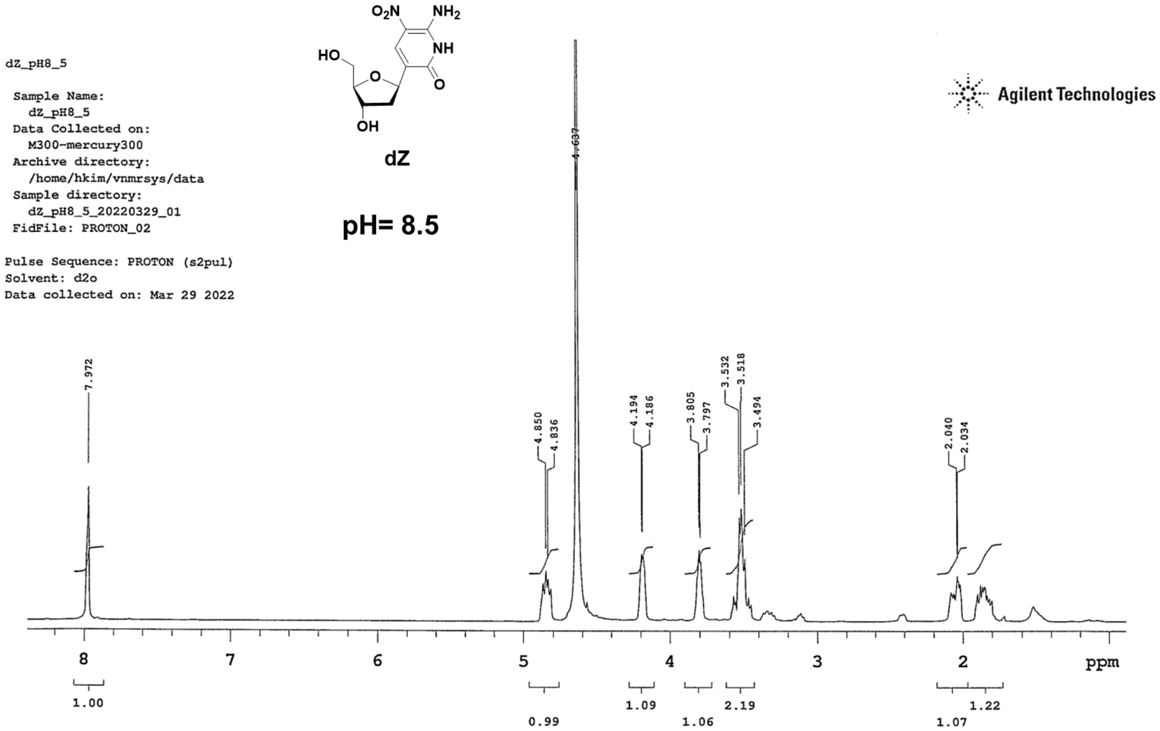
dZ_pH8_5
Sample Name:
dZ_pH8_5
Data Collected on:
M300-mercury300
Archive directory:
/home/hkim/vnmrsys/data
Sample directory:
dZ_pH8_5_20220329_01
FidFile: PROTON_02
Pulse Sequence: PROTON (s2pul)
Solvent: d2o
Data collected on: Mar 29 2022
O_2N
NH_2
NH
HO
O
OH
dZ
pH= 8.5
Agilent Technologies
7.972
4.850
4.836
4.637
4.194
4.186
3.805
3.797
3.532
3.518
3.494
2.040
2.034
8
7
6
5
4
3
2
ppm
1.00
0.99
1.09
1.06
2.19
1.07
1.22

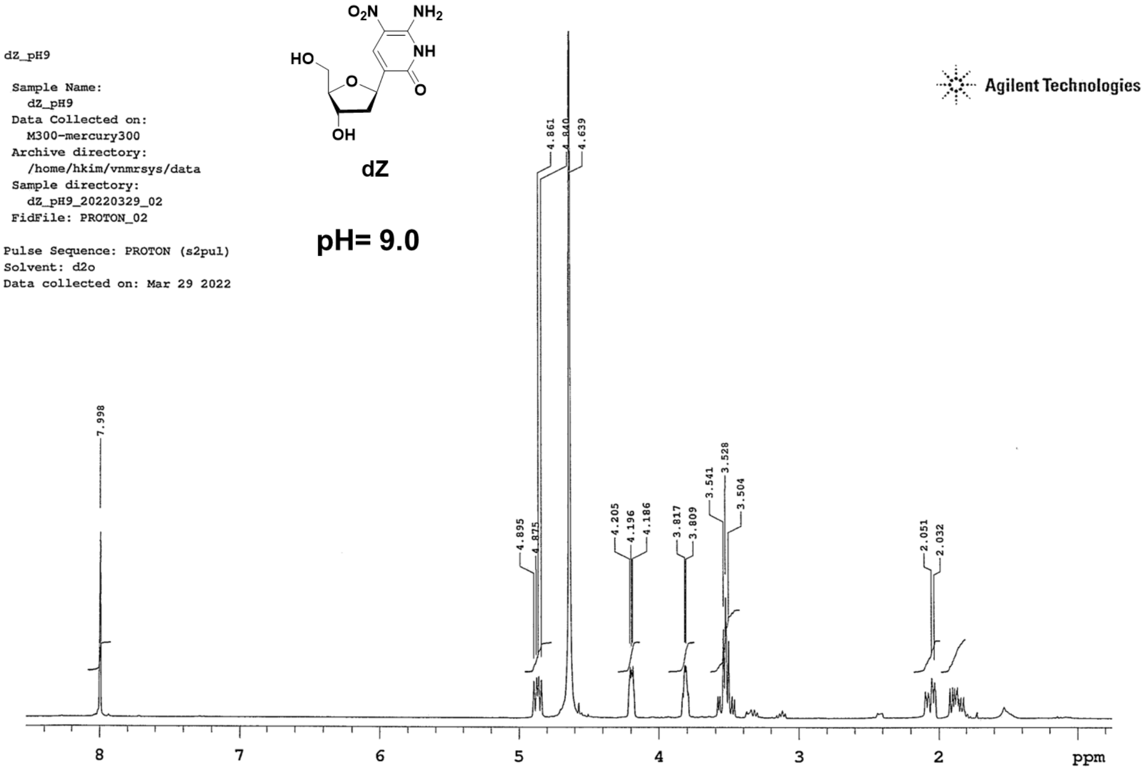
dZ_pH9
Sample Name:
dZ_pH9
Data Collected on:
M300-mercury300
Archive directory:
/home/hkim/vnmrsys/data
Sample directory:
dZ_pH9_20220329_02
FidFile: PROTON_02
Pulse Sequence: PROTON (s2pul)
Solvent: d2o
Data collected on: Mar 29 2022
O_2N
NH_2
NH
HO
O
OH
dZ
pH= 9.0
Agilent Technologies
7.998
4.895
4.875
4.861
4.840
4.639
4.205
4.196
4.186
3.817
3.809
3.541
3.528
3.504
2.051
2.032
8
7
6
5
4
3
2
ppm

References

1. Matsuura, M.F., Kim, H.J., Takahashi, D., Abboud, K.A. & Benner, S.A. Crystal structures of deprotonated nucleobases from an expanded DNA alphabet. *Acta Crystallogr C Struct Chem* **72**, 952-959 (2016).
2. Barker, D.L. & Marsh, R.E.J.A.C. The crystal structure of cytosine. *Acta Cryst.* **17**, 1581-1587 (1964).
3. Murata, T., Enomoto, Y. & Saito, G. Exploration of charge-transfer complexes of a nucleobase: Crystal structure and properties of cytosine–Et2TCNQ salt. *Solid State Sciences* **10**, 1364-1368 (2008).
