# Supplementary figures and images for: A folding motif formed with an expanded genetic alphabet
Source: Nat Chem. 2024 Jun 10;16(10):1715–22. doi: 10.1038/s41557-024-01552-7 (PMC11446821; doi:10.1038/s41557-024-01552-7)

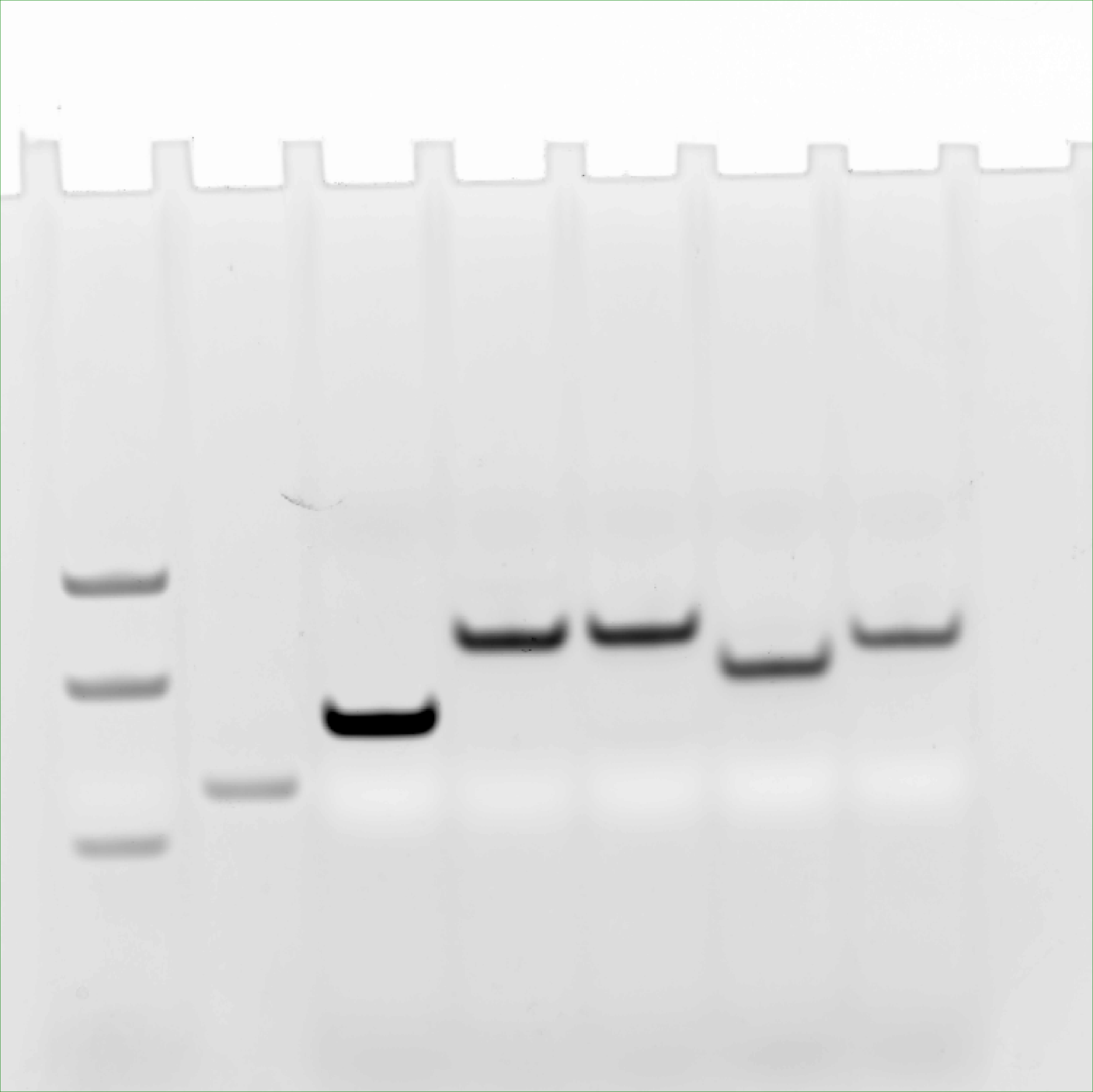

Supplement: Supplementary file 3 — Statistical source data for Fig. 2 and full-length, unprocessed gels for Fig. 2d–f. [file 41557_2024_1552_MOESM3_ESM.zip › SourceData_Fig2/gel picture Fig 2d.tif]

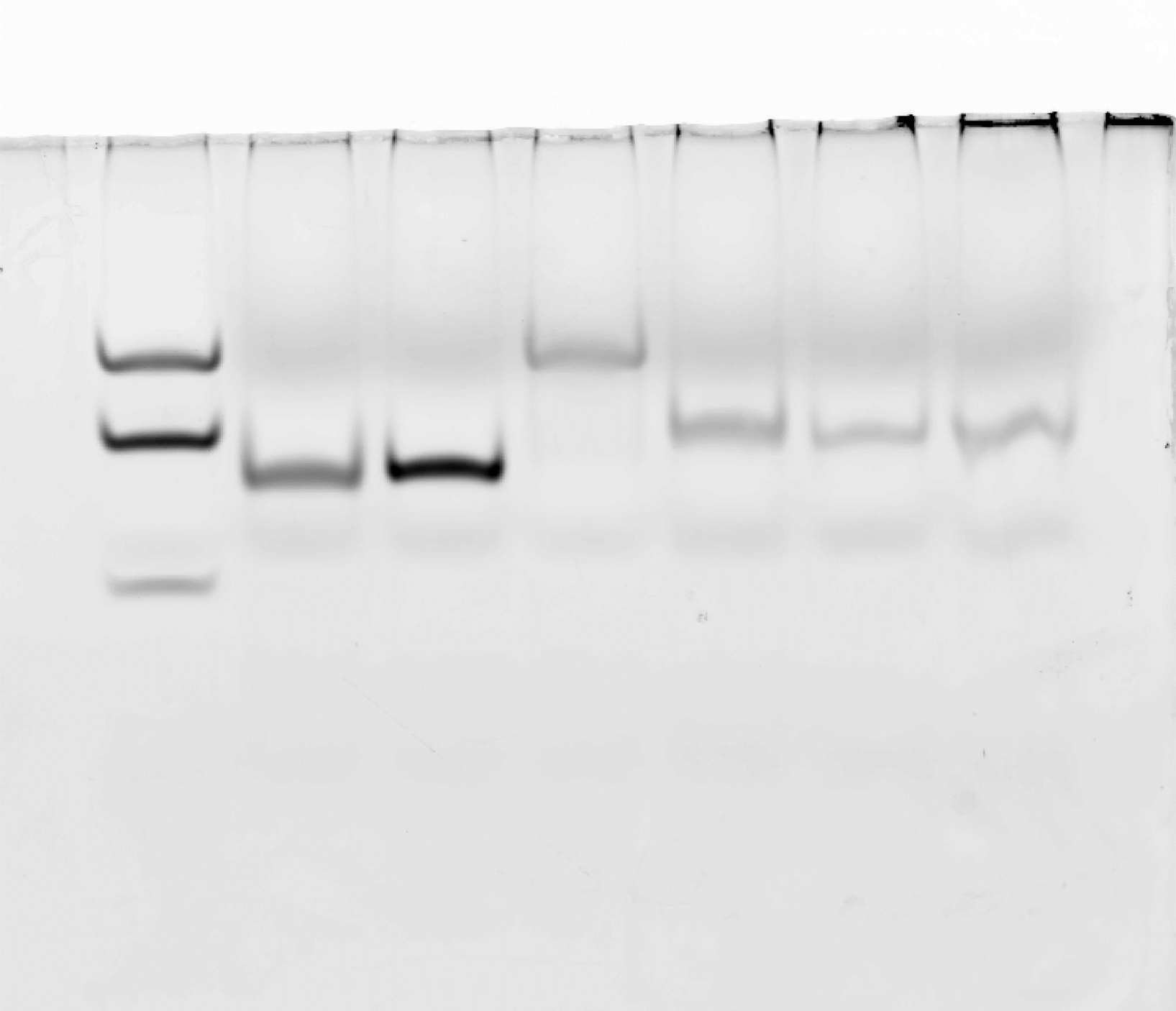

Supplement: Supplementary file 3 — Statistical source data for Fig. 2 and full-length, unprocessed gels for Fig. 2d–f. [file 41557_2024_1552_MOESM3_ESM.zip › SourceData_Fig2/gel picture Fig 2e.tif]

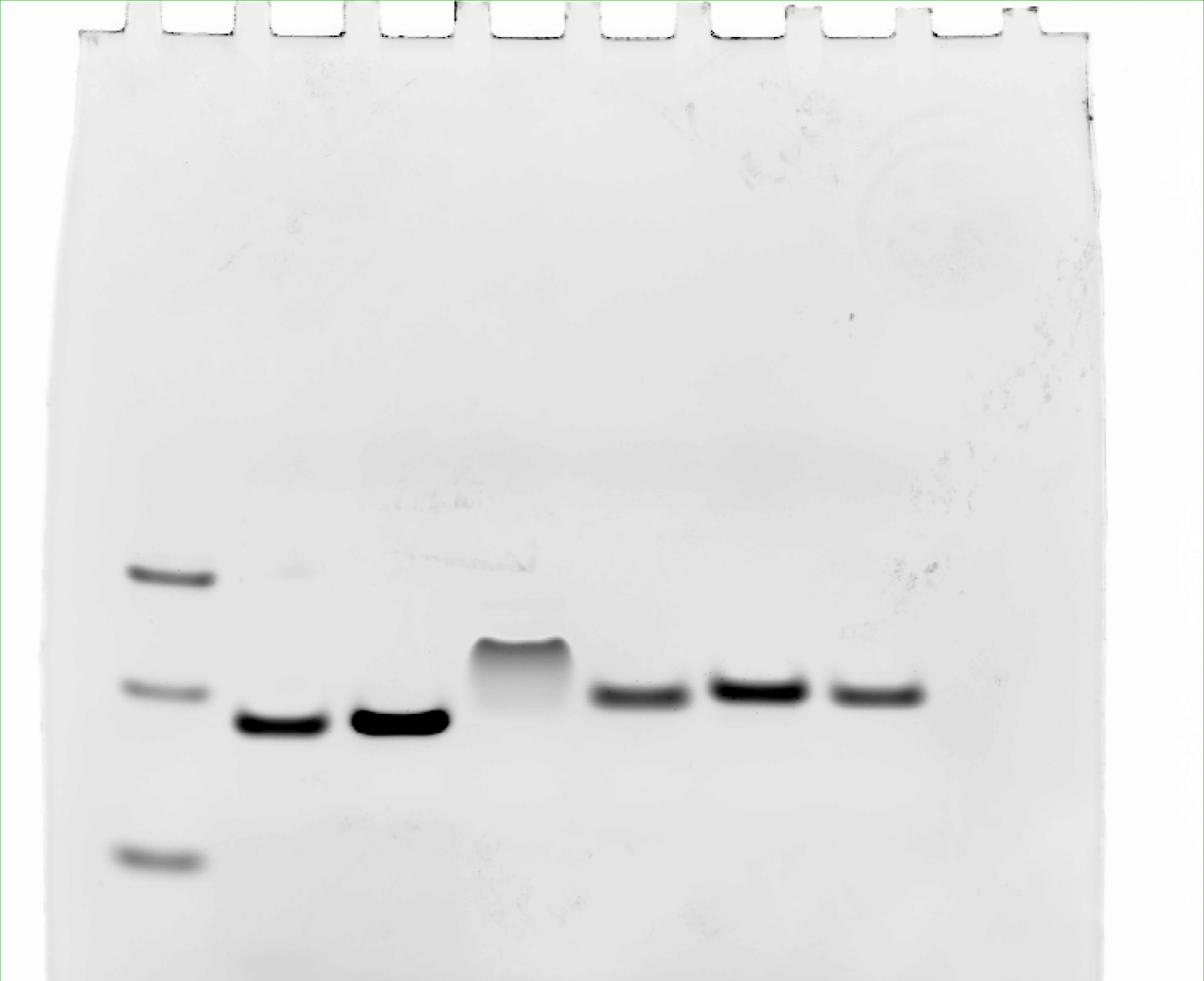

Supplement: Supplementary file 3 — Statistical source data for Fig. 2 and full-length, unprocessed gels for Fig. 2d–f. [file 41557_2024_1552_MOESM3_ESM.zip › SourceData_Fig2/gel picture Fig 2f.tif]
